# The Location of the Antimicrobial Peptide Maculatin 1.1 in Model Bacterial Membranes

**DOI:** 10.3389/fchem.2020.00572

**Published:** 2020-07-07

**Authors:** Anton P. Le Brun, Shiying Zhu, Marc-Antoine Sani, Frances Separovic

**Affiliations:** ^1^Australian Centre for Neutron Scattering, Australian Nuclear Science and Technology Organisation, Sydney, NSW, Australia; ^2^School of Chemistry, Bio21 Institute, University of Melbourne, Melbourne, VIC, Australia

**Keywords:** antimicrobial peptide, bicelles, neutron reflectometry, paramagnetic relaxation enhancement, solid-state NMR, solution NMR, molecular dynamics, mode of action

## Abstract

Maculatin 1.1 (Mac1) is an antimicrobial peptide (AMP) from the skin secretions of Australian tree frogs. In this work, the interaction of Mac1 with anionic phospholipid bilayers was investigated by NMR, circular dichroism (CD) spectroscopy, neutron reflectometry (NR) and molecular dynamics (MD). In buffer, the peptide is unstructured but in the presence of anionic (DPC/LMPG) micelles or (DMPC/DMPG/DHPC) bicelles adopts a helical structure. Addition of the soluble paramagnetic agent gadolinium (Gd-DTPA) into the Mac1-DPC/LMPG micelle solution showed that the N-terminus is more exposed to the hydrophilic Gd-DTPA than the C-terminus in micelles. ^2^H and ^31^P solid-state NMR showed that Mac1 had a greater effect on the anionic lipid (DMPG). A deuterium labeled Mac1 used in NR experiments indicated that the AMP spanned across anionic (PC/PG) bilayers, which was compatible with MD simulations. Simulations also showed that Mac1 orientation remained transmembrane in bilayers and wrapped on the surface of the micelles regardless of the lipid or detergent charge. Thus, the peptide orientation appears to be more susceptible to curvature than charged surface. These results support the formation of transmembrane pores by Mac1 in model bacterial membranes.

## Introduction

Maculatin 1.1 (Mac1)s is an antimicrobial peptide (AMP) from the skin secretions of the Australian tree frog *Litoria genimaculata* (Rozek et al., 1998). The peptide forms part of the frog's innate immune system and is effective at killing a wide range of Gram-positive bacteria (Fernandez et al., 2009). This makes Mac1, along with a range of other AMPs found across nature, a possibility for development of alternative antibiotics (Lee et al., 2015). Alternatives are required due to the increasing prevalence of antibiotic resistance by bacteria to the most commonly used antibiotics currently available, making what were once treatable infections increasingly difficult to treat (Rice, 2009). Mechanistically, what makes most AMPs an attractive target is that they target disrupting the cell membrane rendering the cell unviable and making it improbable for resistance to occur, rather than targeting a metabolic process where bacteria can evolve to develop resistance (Lee et al., 2019).

Bacteria are broadly classified as either Gram-positive or Gram-negative based on the difference in the cell envelopes and their membranes consist of at least 15% anionic lipids (Epand and Epand, 2009). Membrane-active AMPs usually disrupt bacterial membranes in one of three ways: lysis of the membrane through an action known as the carpet mechanism, formation of a toroidal pore or formation of a barrel-stave pore (Sani and Separovic, 2016). The mode of action of AMPs is normally determined by the presence of positively charge residues, amphiphilicity, secondary structure, and lipid composition and charge of the target cell membrane (Koehbach and Craik, 2019). Previous investigations found that AMPs severely perturb anionic bacterial membranes but are less active against eukaryotic membranes (Balhara et al., 2013; Lee et al., 2013). The first driver of AMPs to approach a target cell membrane is the electrostatic interaction between the positively charged residues of the peptide and the negatively charged cell surface. The second driver is then the hydrophobic interactions between the amphipathic domains of the peptide and the acyl chains of the lipids that make up the membrane (Brogden, 2005). As peptides increase in length, net charge and/or hydrophobicity, the ability to disturb anionic lipid systems also increases (Jiang et al., 2008; Lee et al., 2014). Mac1 is 21 amino acids long and is disordered in aqueous solution but when bound to a membrane can span both leaflets of the membrane and adopt an alpha-helix structure (Chia et al., 2000; Fernandez et al., 2013a). At neutral pH Mac1 is cationic so has a strong affinity for anionic membranes such as bacterial membranes but will still bind to zwitterionic membranes (Mechler et al., 2007). The lipid tail composition as well as headgroup makeup will also influence Mac1 membrane binding with a preference for fluid phospholipid bilayers with alkyl chains that are 14 to 16 carbons in length (Sani et al., 2012; Lee et al., 2018). The final characteristic to note is that Mac1 has a proline residue that induces a kink in the structure of the peptide, and substitution of this residue with alanine or glycine results in reduced binding to membranes (Fernandez et al., 2013b). This is the result of the proline creating a wedge which allows the peptide to insert into the bilayer (Sani et al., 2015b).

Determining the structure and orientation of peptides bound within lipids is challenging due to the difficulty in having a single stable conformation of the peptide-lipid aggregate. A number of studies have investigated the location and orientation of Mac1 in lipid bilayers of various lipid compositions and a range of biomimetic membranes. Earlier work showed that Mac1 inserts into the hydrophobic core of the membrane at an angle of approximately 30° to 40° from the bilayer normal, depending on peptide concentration and lipid composition used, and with anionic lipids showing larger tilt angles (Chia et al., 2002; Marcotte et al., 2003). However, from this work, the location of Mac1 could not be ascertained. Previous molecular dynamics (MD) simulations concluded that Mac1 could have multiple insertion angles over a large range (0° to 150°) but clusters of multiple peptides arranged in a parallel fashion in planar bilayers of dipalmitoylphosphatidylcholine, with the N-terminus first making contact with the outer leaflet to facilitate further insertion (Bond et al., 2008). Further MD simulations in an anionic POPE/POPG phospholipid system showed that maculatin spans the lipid bilayer in a parallel fashion but, although a higher density of the N-terminus was found in one leaflet, it was not sufficiently high enough to conclude a preferred orientation (Balatti et al., 2018). Other studies also found that Mac1 can adopt several different aggregate arrangements with dimers to pentamers dominant in phospholipids with short chains, such as DMPC, and hexamers or more dominating in lipids with longer chains (Wang et al., 2016). In the aggregates a mix of anti-parallel and parallel arrangements could be adopted but with the anti-parallel form dominating (Wang et al., 2016). In previous experimental and MD simulation work we have found that Mac1 adopts a preferred orientation in zwitterionic phosphatidylcholine (PC) membranes with the N-terminus in the outer leaflet (Sani et al., 2020). Mac1 will bind to a range of different model membranes that replicate various characteristics of the Gram-positive bacterial membrane (Sani et al., 2015a), and in this study we investigate the orientation of Mac1 in anionic phosphatidylglycerol (PG) membranes that better reflect the charge state of bacterial membranes which is the principal target of Mac1. We use neutron reflectometry and solid-state NMR spectroscopy to determine the location of deuterated Mac1 in PC/PG bilayers that better mimic the properties of membranes of Gram-positive bacteria.

## Materials and Methods

### Materials

Lipids were purchased from Avanti Polar Lipids (Alabaster, USA) and were used without further purification. Lipids used were: 1,2-dimyristoyl-*sn*-glycero-3-phosphocholine (h-DMPC), 1,2-dimyristoyl-*sn*-glycero-3-phospho-(1′-*rac*-glycerol) (h-DMPG), 1,2-dimyristoyl-d54-sn-glycero-3-phosphocholine (d_54_-DMPC), and 1,2-dimyristoyl-d54-*sn*-glycero-3-phospho-(1′-*rac*-glycerol) (d_54_-DMPG), with the d54 in d_54_-DMPC and d_54_-DMPG denoting that the myristoyl chains are labeled with deuterium. Two types of labeled Mac1 were used in this work: ^15^N-labeled Mac1 (GLFGVLAKVAAHVVPAIAEHF-NH_2_), made by solid phase peptide synthesis (in-house facility, Bio21 Institute, Melbourne, Australia) and ^2^H-labeled Mac1 (abbreviated to d-Mac1) (GLFGVLAKVAAHVVPAIAEHF-NH_2_), was used for neutron reflectometry (NR) experiments and was also made in-house by solid-phase synthesis. For the d-Mac1 only the methyl hydrogens of side chains were deuterated. The underlined amino acids in each case indicate which residues were labeled. Deuterated dodecylphosphocholine (d_38_-DPC), deuterated sodium dodecyl sulfate (d_25_-SDS), lysomyristoylphosphatidylglycerol (LMPG) and all other chemicals were purchased from Sigma-Aldrich (Castle Hill, Australia). Silicon wafers were purchased from El-Cat Inc. (Ridgefield Park, USA).

### Solution NMR Experiments

The solution NMR samples were made of: 1 mM multiple (x 11) ^15^N-labeled Mac1 with 98% purity, dissolved in phosphate buffer (10 mM, pH 7.4) containing 50 mM KCl, 1 mM EDTA and 10% (v/v) D_2_O with 120 mM of d_38_-DPC/LMPG (9:1 mol/mol) and 1 mM unlabelled Mac1 in phosphate buffer containing 150 mM d_25_-SDS micelles.

^1^H-^15^N HSQC spectra of ^15^N labeled Mac1 were acquired at 35°C using a Bruker 600 MHz NMR spectrometer for experiments performed with d_38_-DPC/LMPG micelles. For each time increment, 16 transients were taken with 4k and 512 points in the ^1^H and ^15^N dimensions, respectively, and a 1.5 s recycle delay. Spectral width of 13 ppm centered at 4.7 ppm for ^1^H and 40 ppm centered at 118 ppm for ^15^N were used. ^1^H-^15^N-^1^H HSQC-NOESY were performed with same parameters as HSQC experiments and with 4k, 64 and 128 points in the direct and indirect dimensions. For gadolinium titration, a concentrated stock solution of gadolinium diethylenetriamine-pentaacetic acid (Gd-DTPA) in MilliQ water was made and aliquots added directly into the NMR tube to reach 1 mM, 4 mM, 7 mM and 15 mM Gd^3+^. The total added volume of Gd^3+^ was *ca*. 5% of the total sample volume. The NMR tube was allowed to equilibrate for 30 min at 35°C prior to each measurement. The volume of each resonance after Gd-DTPA addition was obtained by integration, corrected for effect of dilution and normalized relative to the Gd^3+^-free resonance volume.

The unlabelled Mac1 in d_25_-SDS micelles sample was investigated at 37°C on an 800 MHz Bruker Advance II spectrometer. ^1^H homonuclear TOCSY (mixing time τ_mix_ = 80 ms) and NOESY (τ_mix_ = 150 and 300 ms) were acquired with 512 points and 1k points in the F1 dimension, respectively, and 4k points in the F2 dimension. Between 16 and 32 transients were accumulated with a 1.5 s recycle delay. The data were multiplied with a squared sine bell function shifted by 90°. The ^1^H spectral window was set to 9,600 Hz. ^13^C-^1^H HSQC experiments were performed with 256 points in the F1 dimension and 4k points in the F2 dimensions. 64 transients were accumulated with a 2 s recycle delay. The ^13^C spectral window was set to 33,200 Hz. Non-uniform sampling ^15^N-^1^H HSQC experiments were performed with 37.5% of 128 points in the F1 dimension and 4k points in the F2 dimension. 1,024 Transients were accumulated with a 1.5s recycle delay. The ^15^N spectral window was set to 3,240 Hz.

All data dimensions were zero-filled to twice the respective FID size. ^1^H chemical shifts were referenced to DSS (sodium trimethylsilylpropanesulfonate) at 0 ppm. Data were processed in Topspin (Bruker) and analyzed using the CCPNmr Analysis program (Vranken et al., 2005). Backbone and side chains were assigned using all experiments.

#### Structure Calculations

The NOESY cross-peak assignments were subsequently used to generate distance restraints for the structure determination. The nOe distance restraints were supplemented with dihedral angle restraints predicted with DANGLE from H_α_, H_N_, N_H_, C_α_, C_β_ chemical shifts (Cheung et al., 2010). A standard CNS 1.1-based protocol was employed using the ARIA 2.2 interface (Rieping et al., 2006). The 10 lowest energy structures were refined in a water shell and evaluated with MolProbity (Chen et al., 2010).

### Solid-State NMR Experiments

The solid-state NMR sample consisted of the unlabelled Mac1 dissolved in anisotropic bicelles at a lipid to peptide molar ratio of 50:1. The anisotropic bicelles were composed of either d_54_-DMPC and DMPG, or DMPC and d_54_-DMPG, at a molar ratio of 4:1 and DHPC lipids mixed at a molar ratio (DMPC+DMPG)/DHPC (q) of 3.6. The lipid concentration (C_L_) was 20% (w/v) in imidazole buffer. The sample was then packed into a 4 mm Bruker MAS rotor.

#### ^31^P NMR Experiments

^31^P NMR experiments were performed on a 400 MHz Bruker Avance III NMR spectrometer at a frequency of 161.5 MHz. A 4 mm triple resonance probe was used in a double resonance mode. 62.5 kHz direct excitation pulse experiments were used under 31.25 kHz ^1^H SPINAL64 decoupling scheme and a recycle delay of 3 s. Typically, 1k scans were acquired and processed with 8k zero-filling and linebroadening from 20 Hz to 100 Hz were used.

#### ^2^H NMR Experiments

The static ^2^H solid-state NMR experiments were performed on a 400 MHz Bruker Avance III NMR spectrometer at a frequency of 61.5 MHz. A 4 mm triple resonance probe was used in a double resonance mode. The solid echo pulse sequence was used with 45.5 kHz ^2^H excitation, an echo delay of 26 μs and a recycle delay of 0.5 s. The spectra were recorded with a 500 kHz spectral window and typically 128 k transients were accumulated. The FIDs were processed using 4 k zero filling and a 150 Hz line broadening.

### Circular Dichroism Experiments

The CD samples were made with phosphate buffer since imidazole prevents signal acquisition below 210 nm. Similar lipid to peptide molar ratios were used as for NMR. CD spectra were acquired on a Chirascan spectropolarimeter (Applied Photophysics Ltd, UK) between 180 and 260 nm using a 0.1 mm path length cylindrical quartz cell (Starna, Hainault, UK). Spectra were acquired at 1 nm intervals, 1 s integration time and 3 scans accumulated. CD signal was recorded in milli-degree units at 35°C and reported as mean residue ellipticity (MRE) using the conversion formula:

$$\begin{array}{l} MRE=\frac{CD}{\left[Mac1\right]\times 10\times L\times N_{r}} \end{array}$$

where CD is the signal in mdegree, [Mac1] the concentration of peptide in mol.L^−1^, L the cell path length in cm and N_r_ the number of residues.

### Neutron Reflectometry

D_2_O and H_2_O solutions for NR experiments were buffered with 10 mM MOPS pH/D 7.0 and 150 mM NaCl. Samples for NR were prepared on round silicon wafers that were 100 mm in diameter and 10 mm thick. Before use the silicon wafers were cleaned in a Jelight UV-ozone cleaner for 20 min and then extensively washed with 2% (v/v) Hellmanex solution, followed by ultrapure (>18 MΩ) water, and then analytical grade ethanol. The wafers were dried under a stream of nitrogen. The polished side of the wafer was held against a roughened backing silicon wafer that had inlet and outlet holes that were connected to a HPLC pump for solution exchange. There was a 100 μm thick PTFE gasket that separated the sample and backing wafer creating a 283 μL volume for sample/solution injection. The wafers were held together in aluminum clamping plates that were also connected to a Julabo water bath for temperature control. Solid-supported membranes were created through vesicle deposition as described previously (Fernandez et al., 2013a) by injecting a 0.1 mg mL^−1^ vesicle solution in the reservoir, incubating for 1 h at 30°C and then rising excess vesicles away with 5 mL of buffer. After characterization of the membranes 2 mL of d-Mac1 at 10 μM was injected and incubated for 1 h before excess peptide was removed with a 5 mL wash of buffer.

Neutron reflectometry measurements were conducted on the blank silicon wafer, the formed solid-supported membrane, and after Mac1 incubation on the Platypus time-of-flight neutron reflectometer at the 20 MW OPAL Research Reactor (Sydney, Australia) (James et al., 2011). The instrument views the cold-neutron source and utilizes a useable wavelength bandwidth of 2.5 to 20 Å using a disc chopper system that was set to a Δλ/λ ~ 8.4% and 24 Hz. The instrument has horizontal sample geometry and collimation slits of 0.71 and 2.91 mm were used for an angle of incidence of 0.85° and 3.5°. Neutrons were detected on a 2D ^3^He detector and the raw data was reduced using in-house software that stitches the two angles together at the appropriate overlap region, re-bins the data to instrument resolution, correctly scales the data so that any critical edge is unity and corrects for detector efficiency (Nelson, 2010). The final data is then presented as reflectivity vs. momentum transfer, Q, which is given according the equation below:

$$\begin{array}{l} Q=\frac{4\pi \sin\theta }{\lambda } \end{array}$$

where θ is the angle of incidence and λ the wavelength.

Data was analyzed using an Abele's matrix method in the MOTOFIT software package (Nelson, 2006). The membrane system is divided into a series of layers and each layer is defined by its thickness (in Å), its scattering length density (SLD) (in Å^−2^) and interfacial roughness (in Å). An initial model is created and then a reflectivity profile calculated and compared against the experimental data. Parameters are then adjusted until the calculated profile adequately matches the experimental data using a least-squares regression. A genetic algorithm is used so that reasonable limits can be placed on each varied parameter and to avoid becoming trapped in global minima. A scattering length density is analogous to a neutron's refractive index and can be calculated according to:

$$\begin{array}{l} SLD=\frac{\sum_{n=1}^{i}b_{i}}{V_{m}} \end{array}$$

where V_m_ is the molecular volume and b_i_ is the scattering length for each isotope within the molecule. The theoretical SLD and molecular volumes of the materials used can be found in Supplementary Table S1. As scattering length varies across each different isotope (e.g., for hydrogen b_H_ = −3.741 × 10^−5^ Å and for its stable isotope deuterium b_D_ = +6.667 × 10^−5^ Å), different components of each layer can be determined through contrast variation and the total SLD of a layer is determined through the sum of the SLD for each component according to:

$$\begin{array}{r} SLD_{layer}=\left(SLD_{lipid}{Ø}_{lipid}\right)+\left(SLD_{peptide}{Ø}_{peptide}\right)+\\ \left(SLD_{solvent}{Ø}_{solvent}\right) \end{array}$$

where Ø is the volume fraction. The phase of the lipid bilayer at the different temperatures was determined through calculating the area per lipid, A_lipid_, using the fitted properties of the lipid tails as follows:

$$\begin{array}{l} A_{lipid}=\frac{V_{m}}{\tau {Ø}_{lipid}} \end{array}$$

where τ is the thickness of the lipid tails. Error values for each fitted parameter were determined using Monte Carlo resampling as described previously (Heinrich et al., 2009; Holt et al., 2009). Briefly, 1008 fits of the data were completed and the distribution of fitted values plotted for each parameter varied. The error is then the 95% confidence interval of the distribution.

### Molecular Dynamics

The starting conformation of Mac1 peptide was generated from NMR data. The CHARMM-GUI membrane builder (Jo et al., 2007, 2008, 2009; Wu et al., 2014) was then used to prepare the peptide-bilayer system with 40 DMPC and 10 DMPG lipids per leaflet and the peptide-micelle complex with 58 DPC and 7 LMPG lipid molecules randomly distributed. Each system was generated using a rectangular box containing 50 mM KCl and a 12.5 Å layer of water. All histidine residues were singly protonated to model the ionization state expected at pH 7.4 and the peptide C-terminus was amidated to match the experimental conditions. The simulations were performed using the CHARMM-36m force fields. The minimization, equilibration and production runs were performed with the NAMD package on a desktop machine fitting a GPU GeForce GTX 1080 titanium and a CPU with 12 cores.

Each system was first minimized for 2500 steps using the steepest descent method followed by 2500 steps of the conjugate gradient method with a 12 Å non-bonded interaction cut-off. The peptides and lipids were restrained with a 10 and 2.5 kCal.mol^−1^ potential, respectively. Then, 25 ps equilibration MD simulations were run at 308 K for each system, using a 10, 5, 2.5 kCal/mol restraint to maintain the peptide backbone and 2.5, 0 and 0 kCal/mol restraint on the lipids atom positions with 0.001 ps time step. Next, the peptide positional restraints were reduced to 1, 0.5, and 0.1 kCal/mol for 100 ps with 0.002 ps time step. All covalent bonds involving hydrogen atoms were constrained using the SHAKE algorithm (Ryckaert et al., 1977) and the rigid internal geometry for TIP3P water molecules was constrained with the SETTLE algorithm (Miyamoto and Kollman, 1992). The system temperature was maintained at 308 K using a Langevin thermostat (Izaguirre et al., 2001) with a 1 ps^−1^ collision frequency. The system pressure was controlled at 1 bar using a Langevin barostat for the DPC/LMPG micelle and a semi-isotropic Berendsen barostat with a xy surface tension for the DMPC/DMPG bilayer. Finally, the systems were run for 1 ns prior the production runs. 100 ns long simulations were performed with a random restart every 1 ns, the trajectories were then concatenated together, and the analysis was performed on the full 100 ns simulation.

The MD trajectories were visualized and analyzed using VMD (Humphrey et al., 1996) with custom scripts and the CPPTRAJ (Roe and Cheatham, 2013) software and fitting procedures and plots were created in Gnuplot.

## Results

### Effect of Negatively Charged Membranes on Mac1 Helical Conformation and Preferential C-Terminus Insertion

**Figure 1:**
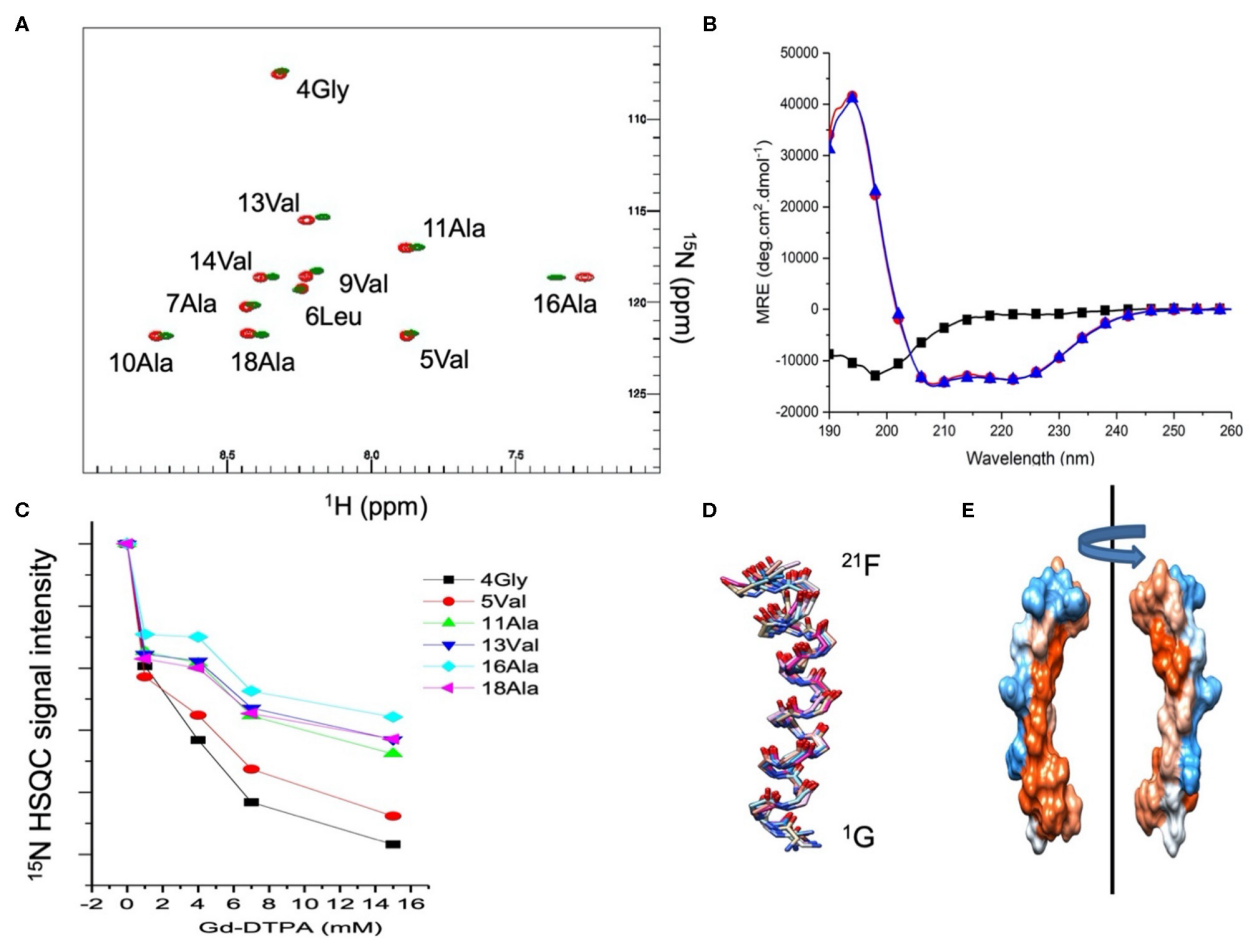
**(A)** ^15^N HSQC spectra of 1 mM Mac1 in the presence of zwitterionic d_38_-DPC micelles (green) or anionic d_38_-DPC/LMPG (9:1) micelles (red). Assignments were made using TOCSY and NOESY experiments and previously published data (Sani et al., 2020). The NMR experiments were performed at 35°C in imidazole buffer (pH 7.4). **(B)** CD spectra of Mac1in aqueous buffer (black squares), in the presence of zwitterionic DPC micelles (blue triangles) or anionic SDS micelles (red circles). The CD experiments were performed at 37°C in phosphate buffer (pH 5). **(C)** Resonance peak intensity of ^15^N labeled Mac1 residues as a function of the Gd-DTPA concentration in the presence of d_38_-DPC/LMPG (9:1) micelles. The NMR experiments were performed at 35°C in imidazole buffer (pH 7.4). **(D)** 10 lowest energy structures of Mac1 calculated from the solution NMR experiments (Supplementary Figure S1 and Supplementary Table S2) performed in SDS micelles (pH 5) at 37°C. **(E)** Electrostatic surface representation of Mac1 averaged structure with the hydrophobic (red), hydrophilic (blue) and neutral (white) residues according to Kyte and Doolittle (1982).

The effect of the membrane curvature on the secondary structure and insertion of Mac1 has been recently characterized in neutral membrane mimetics using solution NMR and CD techniques (Sani et al., 2020). Here, similar investigations were performed in anionic membrane mimetics to probe the role of electrostatic interactions in AMP mode of action. The addition of 10% mol of the anionic LMPG in DPC micelles induced slightly different chemical shift perturbations along the peptide sequence. Comparing the ^15^N HSQC obtained in DPC micelles and DPC/LMPG (9:1) showed that the N-terminus of Mac1 exhibited small chemical shift changes at Val^5^, Leu^6^, and Ala^7^ while the middle and C-terminus sections exhibited more pronounced chemical shift perturbations, the highest observed for Ala16 (Figure 1A). Interestingly, the shielding from the hydrophilic gadolinium moiety was greater for the middle section and the C-terminus while the N-terminus was severely exposed to paramagnetic effects as seen in the signal intensity loss upon Gd^3+^ titration (Figure 1C). The circular dichroism spectra of Mac1 in the presence of DPC and SDS micelles showed small differences: the lineshapes exhibited the typical Hα features of two minima at 222 nm and 209 nm and a maximum at about 195 nm (Figure 1B). The 10 lowest energy structures of Mac1 (Figure 1D) were determined using solution NMR restraints obtained from the NOESY experiments performed in SDS micelles (Supplementary Figure S1). Mac1 showed a continuous Hα stretch with a bend located near Ala16, adjacent to the Pro15 residue. The electrostatic surface of Mac1 was computed using the averaged structure and exhibited an amphipathic distribution. Altogether, these results are consistent with the previously reported structure of Mac1 in DPC micelles and indicate that Mac1 retained a preferential insertion through its C-terminus, despite a slightly greater hydrophilic surface.

### Preferential Interaction of Mac1 With Anionic Lipids

**Figure 2:**
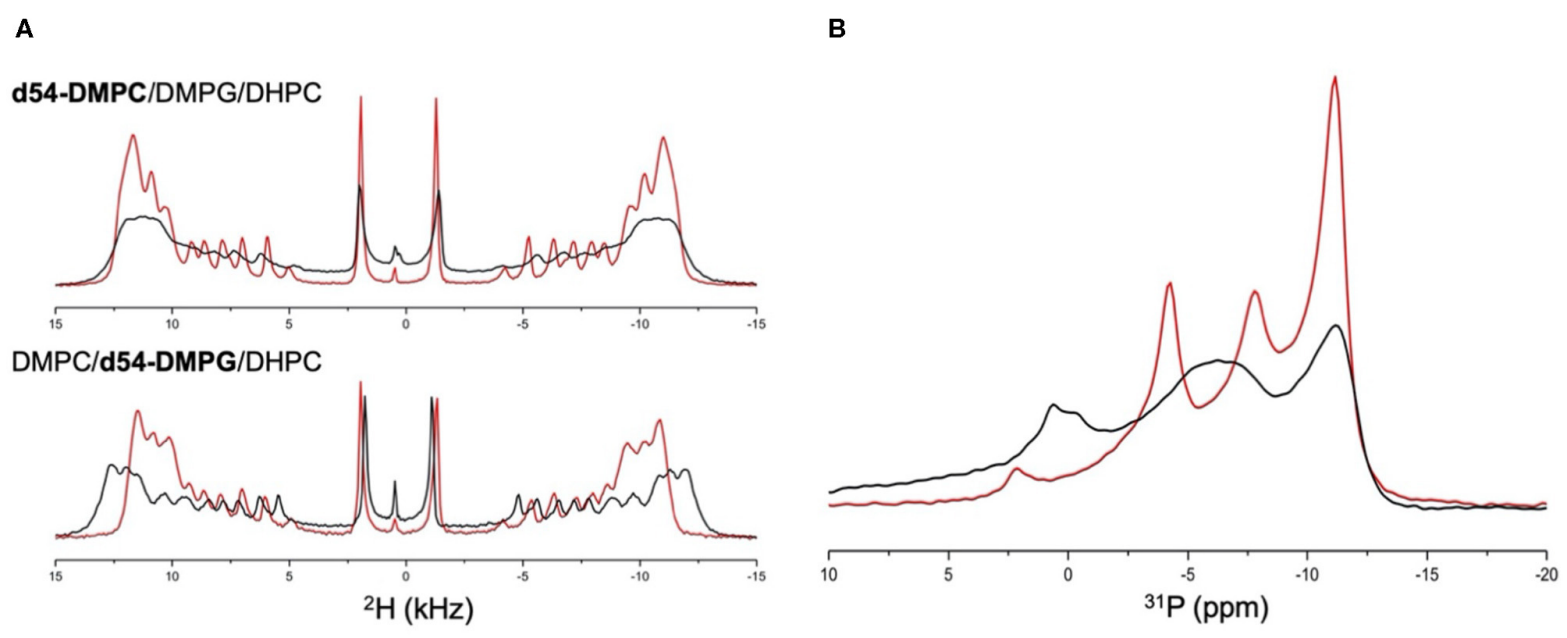
**(A)** ^2^H static NMR spectra of: (top) d_54_-DMPC/DMPG/DHPC anisotropic bicelles (red line) and in the presence of Mac1 (black line); (bottom) DMPC/d_54_-DMPG/DHPC anisotropic bicelles (red line) and in the presence of Mac1 (black line); and **(B)** ^31^P static NMR spectra of d_54_-DMPC/DMPG/DHPC anisotropic bicelles (red line) and in the presence of Mac1 (black line). All experiments were performed at 35°C with a DMPC to DMPG molar ratio of 4:1, a DMPC+DMPG to DHPC molar ratio of 3.6:1 and with a L/P molar ratio of 50:1.

Highly curved surfaces are not optimal to understand the molecular mechanism of interaction between Mac1 and cell membranes. Thus, lipid bilayers of DMPC and DMPG (4:1 mol/mol) were used and anisotropic bicelles were formed using a short chain DHPC lipids (*q* = 3.6). This lipid system increases the spectral resolution due to spontaneous alignment in the magnetic field. By either adding d_54_-DMPC or d_54_-DMPG, specific modulations were observed for neutral or anionic lipids, respectively, and Mac1. The ^2^H spectra displayed in Figure 2A clearly showed that Mac1 decreased strongly the dynamics (or increased the order) of the d_54_-DMPG lipid chains as seen in the increase of the ^2^H quadrupolar splitting. The ^2^H spectrum of the d_54_-DMPC showed little change in the quadrupolar splitting but significant line broadening was observed.

A similar broadening of the overall ^31^P line shape was observed in the presence of Mac1, and interestingly, the ^31^P chemical shift of DMPG and DHPC was significantly shifted while DMPC ^31^P chemical shifts remained mainly unchanged but broadened. This also indicates that PG headgroup is in more curved regions near DHPC; and supports that the cationic Mac1 peptide had a stronger impact on anionic lipids than on the neutral lipids, yet, without heavily disturbing the lipid packing at this lipid to peptide molar ratio.

### Mac1 Bound Within Solid-Supported Phospholipid Membranes

**Figure 3:**
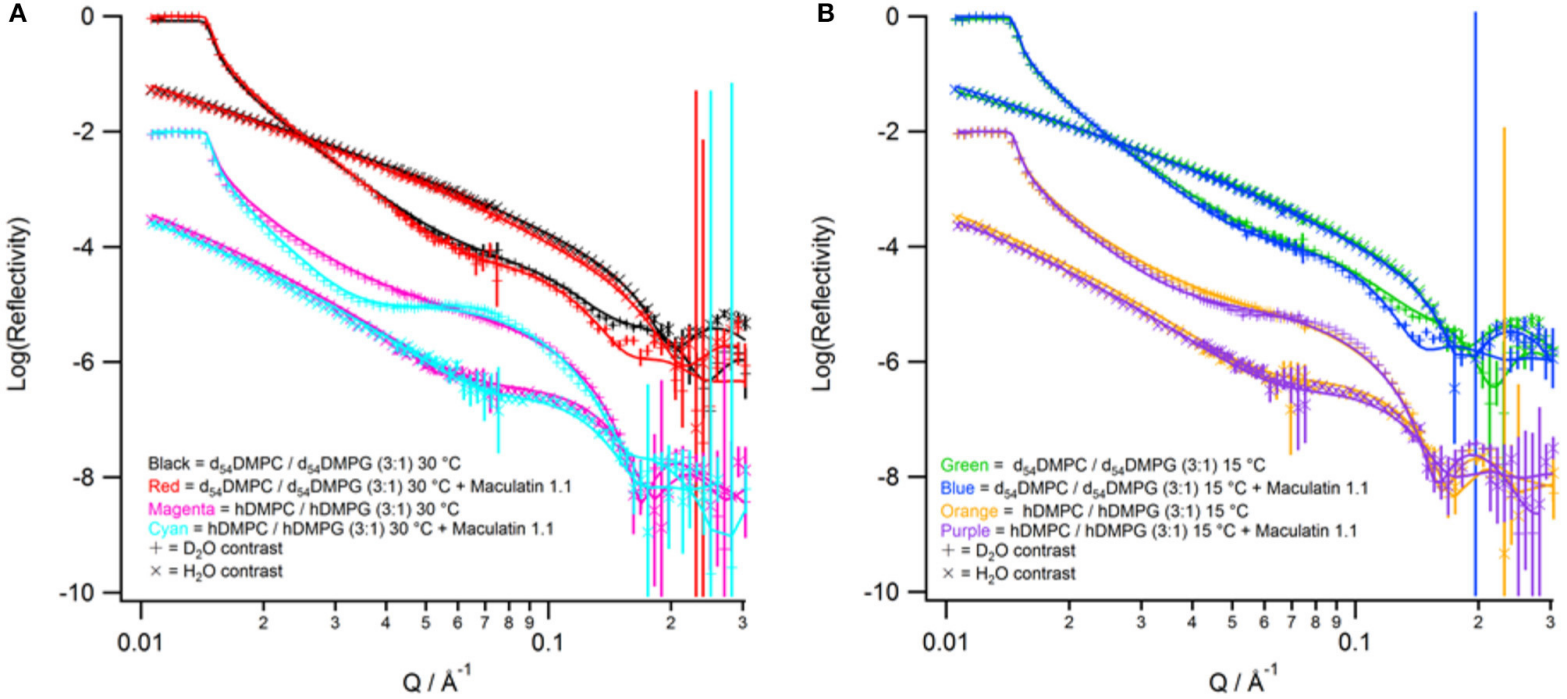
Neutron reflectivity profiles (points with error bars) and fits (solid lines). **(A)** Data collected at 30°C with red for tail-deuterated bilayers only, black as tail deuterated bilayers with Mac1 bound, magenta as hydrogenous bilayers only, and cyan as hydrogenous bilayers with Mac1 present. **(B)** Data collected at 15°C with green for tail-deuterated bilayers only, blue as tail deuterated bilayers with Mac1 bound, orange as hydrogenous bilayers only, and purple as hydrogenous bilayers with Mac1 present. In all cases the D_2_O contrast is represented with + symbols and H_2_O contrasts with × symbols.

Neutron reflectometry is a technique able to probe the structure of surfaces and interfaces at nanoscale dimensions. The information obtained is a one-dimensional description of the structures through the different layers of the system (Penfold and Thomas, 2014). This information is useful for membrane systems as many surface sensitive and microscopy techniques can only view the surface topology whereas NR can probe through the entire bilayer (Wacklin, 2010; Lakey, 2019). The first part of the measurements is creation of a solid-supported phospholipid membrane on a silicon wafer. The membranes, consisting of d_54_-DMPC/d_54_-DMPG or h-DMPC/h-DMPG with a PC to PG mole ratio of 3:1, were deposited using the widely used vesicle deposition technique onto silicon wafers which were found to have a 8.3 ± 3.3 Å and 10.7 ± 2.1 Å oxide layer with a 3.2 Å and 2.6 Å roughness for the deuterated and hydrogenous bilayer, respectively. All reflectometry experiments were characterized in a buffered D_2_O and H_2_O contrast which were fitted simultaneously (Figure 3). For the modeling the bilayer was divided into three separate layers consisting of inner headgroups (those closest to the silicon oxide surface), tails and outer headgroups (those closest to the bulk solvent). The bilayers were characterized at 30°C and were found to have a good coverage with a volume fraction of 0.894 ± 0.133 and 0.965 ± 0.030, and total thicknesses of 52.0 ± 1.7 Å and 59.4 ± 3.6 Å, which corresponds to an area per lipid of 57.4 ± 8.6 Å^2^ and 49.0 ± 1.8 Å^2^ each for the deuterated and hydrogenous bilayers, respectively (Supplementary Table S3). These results are consistent with previous observations of solid-supported membranes with similar lipid composition at 30°C and correspond to being in the liquid crystalline (L_α_) phase (Fernandez et al., 2013a). The bilayers were cooled to 15°C and the bilayer re-measured. Upon cooling the fringe in the reflectivity profile shifts to lower Q-values (Figure 3B) indicating the bilayer has become thicker. After complete data analysis the bilayer properties at 15°C the area per lipid reduces to 45.4 ± 0.8 Å^2^ and 45.0 ± 5.2 Å^2^ for the deuterated and hydrogenous bilayers, respectively (Supplementary Table S3), indicating that the bilayer is now in the gel (L_β_) phase, with the thickness for each bilayer also increasing with the volume fraction remaining largely unchanged (within error). It should be noted that bilayers deposited directly onto solid-supports through methods such a vesicle deposition do not display the ripple (P_β_) phase (Naumann et al., 1992).

**Figure 4:**
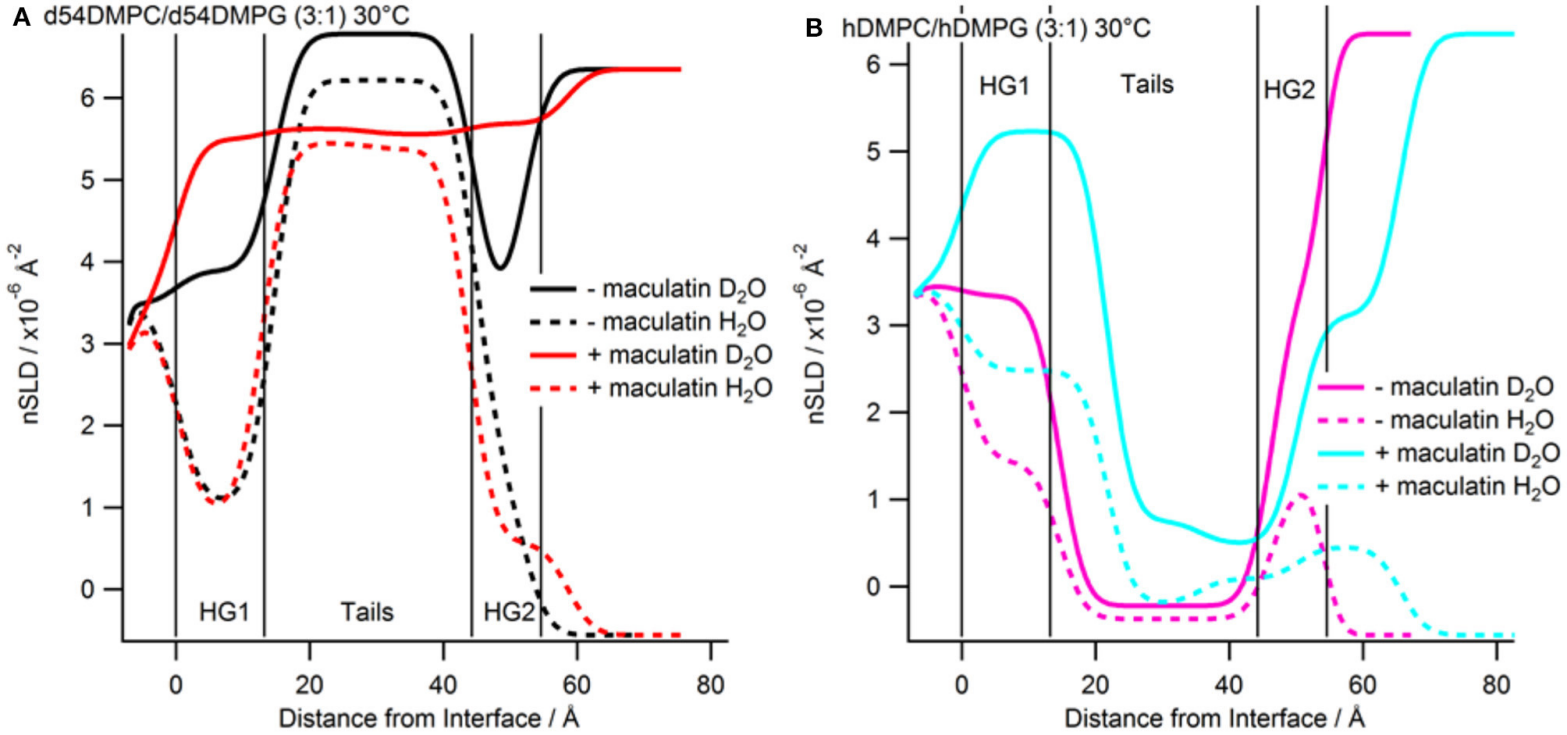
Real-space SLD profiles of the bilayers with and without Mac1 bound at 30°C. **(A)** Tail-deuterated bilayers without (black) and with Mac1 (red) present. **(B)** Hydrogenous bilayers without (magenta) and with Mac1 (cyan). Solid lines are the D_2_O contrast and dashed lines are the H_2_O contrast.

After characterization of the bilayers 2 mL of d-Mac1 prepared in buffered D_2_O at 10 μM was flowed into the sample cell and left to incubate on the bilayer for 1 h at 30°C. After the incubation period the excess peptide was removed with a wash of buffered D_2_O the NR was measured. A version of Mac1 where six of the amino acids in the N-terminal half of the peptide are deuterated creates a substantially different SLD between the N- and C-terminal halves of the peptide (see Supplementary Table S1 and section Materials for details). This means that if Mac1 embeds within the membrane in a specific orientation this will be highlighted as the two leaflets of the membrane will have different SLD values. As can be seen in Figure 3A the reflectivity profiles before and after peptide look different suggesting that d-Mac1 has bound to the bilayer. Initially, the bilayers with peptide bound were fitted using the same three layer model above. However, a four layer model, where the tails were split into two separate layers of inner and outer tails with the SLD allowed to vary for each layer, was more suitable. After peptide addition it was observed that, overall the combined thickness of the tails layers increased in the presence of d-Mac1 (Figure 4 and Supplementary Table S4), which is consistent with previous observations of h-Mac1 binding to anionic lipid bilayers (Fernandez et al., 2013a). The SLD of the tail layer changed in both leaflets with an overall decrease for the deuterated bilayer (Figure 4A) and increase for the hydrogenous bilayer (Figure 4B), which indicated that the d-Mac1 has inserted across both leaflets of the bilayer.

Examining more closely the SLD of each leaflet, a difference between the two sides of the bilayer (Figure 4 and Supplementary Table S5), which is particularly notable in the h-DMPC/h-DMPG (3:1) membrane (Figure 4B), can be seen. One interpretation is that the variation in SLD across the bilayer leaflet after peptide addition is due to an uneven distribution of d-Mac1 across the lipid leaflets. Upon calculating the volume fractions of d-Mac1 using this scenario, the volume fraction for the inner/outer leaflet are 0.172/0.209 and 0.118/0.182 for the deuterated and hydrogenous bilayer, respectively (Supplementary Table S4). However, as d-Mac1 is 21 amino acids and long enough to span a bilayer then, if the peptide is assembling in the lipid bilayer in a parallel fashion, the uneven SLD could be due to a preferred orientation. In most cases the SLD of the inner leaflet is slightly higher than that of the outer leaflet. This would suggest an orientation where the N-terminal half of the peptide is buried in the lower leaflet, which is curiously the opposite orientation of the d-Mac1 in a pure DMPC bilayer where the N-terminal half is found in the outer leaflet (Sani et al., 2020). The volume fraction of peptide in this scenario would be 0.256 for the deuterated bilayer and 0.176 for the hydrogenous bilayer. In either scenario, lipid is lost from the surface suggesting some lytic activity. It should be noted that for the anionic membranes used in this study, the difference in SLD this time is within error of each other and there is also a contribution to the SLD of the layer from the volume fraction of solvent present, which would have an uneven distribution across the two bilayer leaflets.

**Figure 5:**
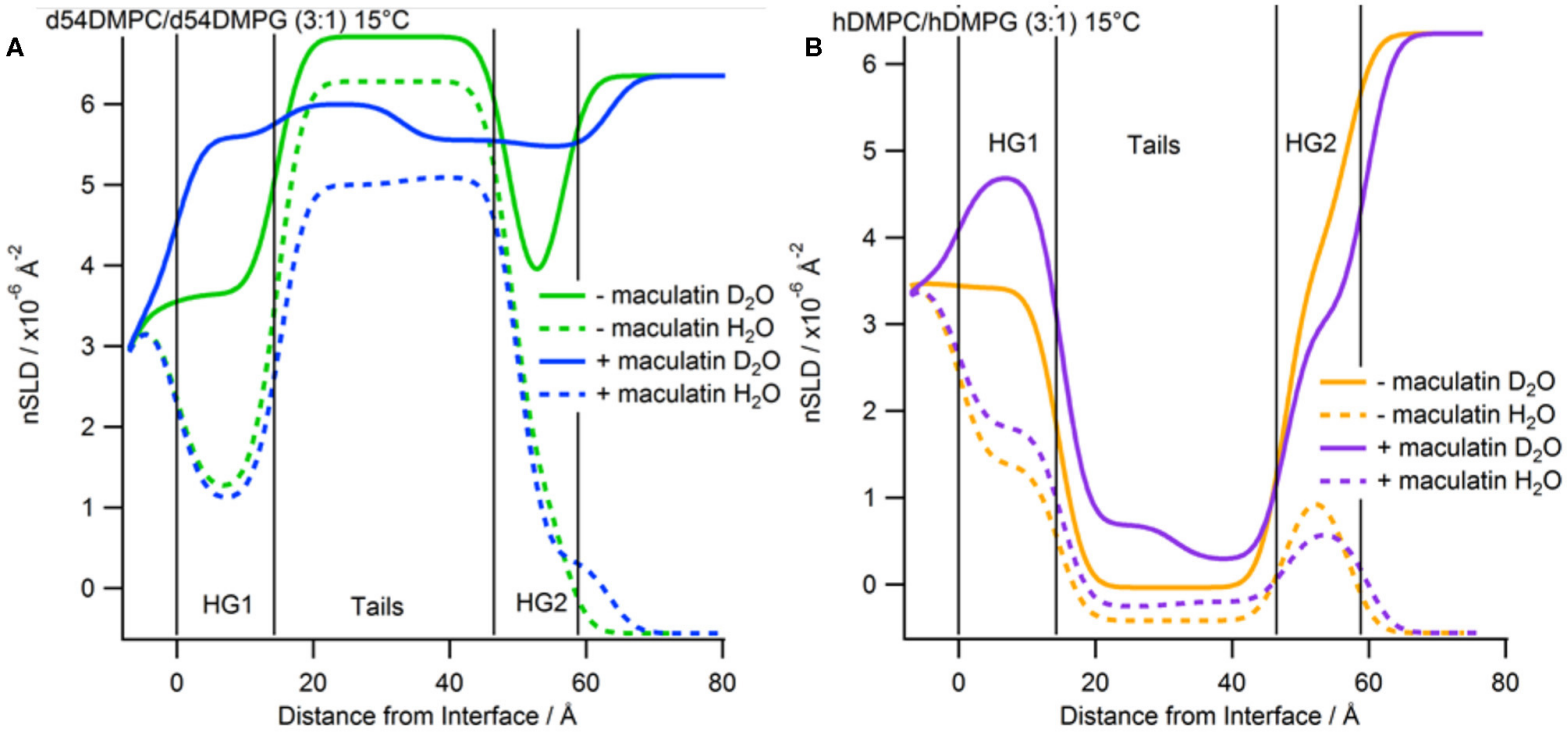
Real-space SLD profiles of the bilayers with and without Mac1 bound at 15°C. **(A)** Tail-deuterated bilayers without (green) and with Mac1 (blue) present. **(B)** Hydrogenous bilayers without (orange) and with Mac1 (magenta). Solid lines are the D_2_O contrast and dashed lines are the H_2_O contrast.

The bilayers with d-Mac1 bound were cooled to 15°C and remeasured. As for peptide-free bilayers the reflectivity shifts to lower Q-values (Figure 3), indicating that the membrane has become thicker. Looking at the values for thickness the bilayer does indeed become thicker with an increase of 2.2 Å (Supplementary Table S4). A difference in SLD across the two leaflets was also maintained showing that changes in the phase state of the membrane for a DMPC/DMPG (3:1) composition is not influenced by the presence of d-Mac1. Additionally, cooling the DMPC/DMPG (3:1) bilayers to the gel phase did not alter the orientation or distribution of d-Mac1 within the bilayer with no change observed in the orientation of the peptide (Figure 5). The difference in SLD in the inner tails increases when the temperature was changed from 30°C to 15°C in the d_54_-DMPC/d_54_-DMPG (3:1) case (Supplementary Table S5) with this difference corresponding to a loss of lipid and, therefore, an increase in the peptide volume fraction (Supplementary Table S4).

### Mac1 Exposure to Solvent Is Dependent on Membrane Curvature Rather Than Surface Charge

Full atom MD simulations were used to gain residue specific details of Mac1 interaction with anionic micelles and bilayers. The initial configurations were set as a single Mac1 peptide fully inserted into a micelle made up of 54 DPC and 6 LMPG or a bilayer made of 40 DMPC and 10 DMPG molecules per leaflet. Each system was equilibrated for *ca*. 1 ns at 35°C as described in the Methods section. The 35°C temperature was chosen as this is well above the phase transition temperature for both lipid types and are thus unlikely to form different phase domains (Lewis et al., 2005).

#### Effect of Anionic Lipids on Mac1 Topology and Length

**Figure 6:**
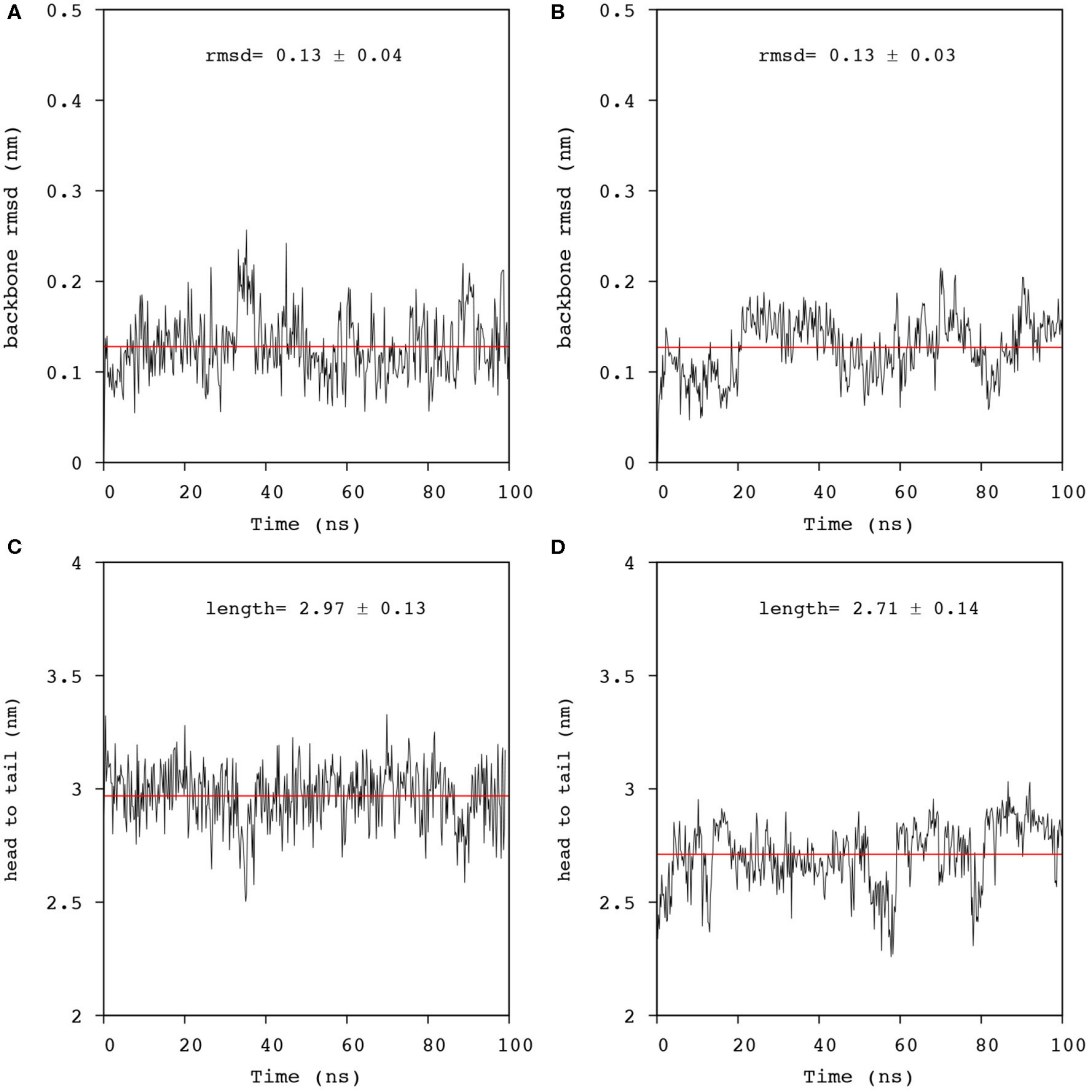
Analysis of the trajectories obtained from 100 ns simulation of Mac1 in lipid environments. N-Cα-C backbone rmsd from the equilibrated structure in: **(A)** a DPC/LMPG (9:1) micelle, and **(B)** a DMPC/DMPG (4:1) bilayer using residues 1-21 (black line). The averaged rmsd was fitted with a linear function (red line). Distance fluctuation between the ^1^Gly and the ^21^Phe nitrogens of Mac1 inserted in: **(C)** a DPC/LMPG (9:1) micelle, and **(D)** a DMPC/DMPG (4:1) bilayer. The averaged distance was obtained by fitting the data to a linear function (red line).

**Figure 7:**
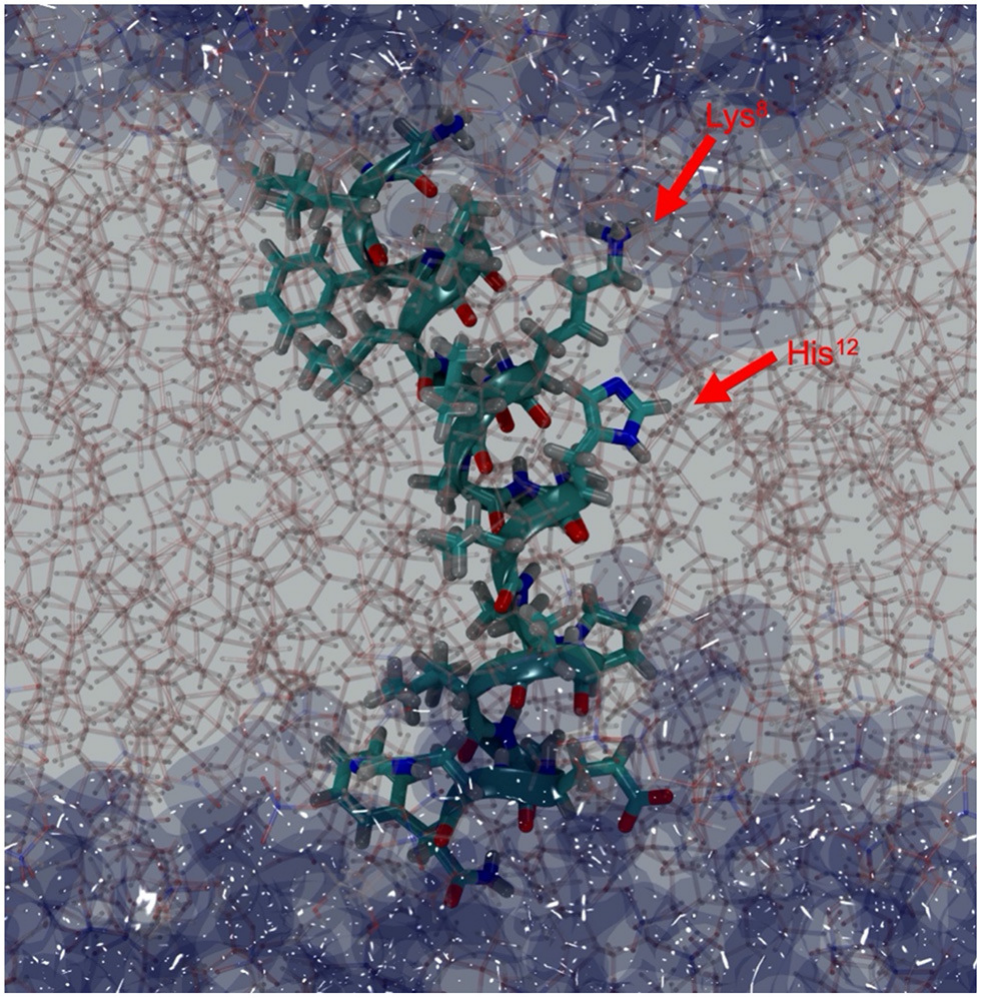
Depth-cued snapshot of Mac1 in DMPC/DMPG (4:1) bilayers (sticks) at 57 ns showing a bent structure with water (dark blue surface) penetrating through the bilayer. Lys^8^ is snorkeling out to reach the water interface and His^12^, Pro^15^/Ala^16^, and Asp^19^ provide the hydrophilic surface for water molecules to hydrate the peptide within the hydrophobic core.

Mac1 migrated to a peripheral location with a wrapped configuration around the micelle but remained in a transmembrane orientation in the bilayer, a similar outcome as for MD simulations performed in neutral lipid systems (Sani et al., 2020). The N-Cα-C backbone structure fluctuations were also similar for both anionic systems and neutral systems across the 100 ns simulations, indicating that Mac1 secondary structure was not greatly different in neutral and anionic lipid systems. Furthermore, the peptide length, measured as the head to tail distance between the nitrogen backbone atoms, (Figure 6) showed little variation in the micelle (about 3 nm) but some fluctuation in the bilayer system were observed with a significantly lower averaged peptide length (2.7 nm). As seen in Figure 7, the difference corresponded to a flexible bend initiated at Val^14^ (before Pro^15^), bringing the lowest peptide length to 2.3 nm, well below the optimal theoretical length for a 21 amino acid long linear peptide in an α-helical configuration of 3.15 nm.

#### Insertion Depth and Water Penetration

**Figure 8:**
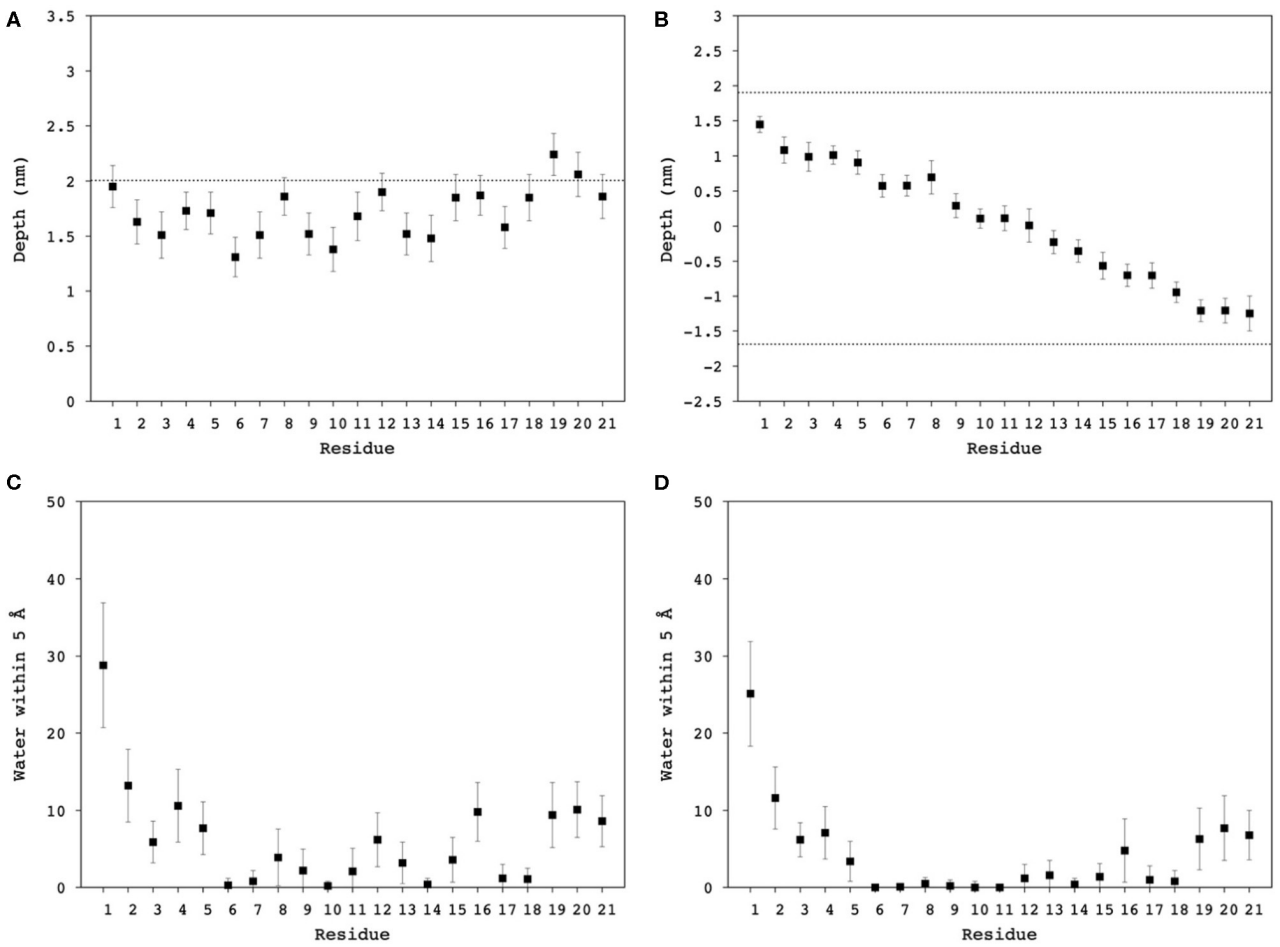
Averaged distance from the N^th^ residue to the center of mass of **(A)** the DPC/LMPG (9:1) micelle, and **(B)** DMPC/DMPG (4:1) bilayer, as a proxy of Mac1 insertion depth into the hydrophobic core of the lipids. Averaged contact number between the n^th^ residue nitrogen and water molecules within 5 Å in **(C)** DPC/LMPG (9:1) micelle, and **(D)** DMPC/DMPG (4:1) bilayer. The dashed lines correspond to the phosphates averaged position to the center of mass of the lipid systems.

The depth of Mac1 penetration into the lipid core (Supplementary Figures S2, S3) and its exposure to water (Supplementary Figures S4, S5) were calculated over the 100 ns simulations. On average, Mac1 was positioned mainly just below the phosphates in the micelle (Figure 8A), the middle section of the peptide (from Leu^6^ to Val^14^, excepting Lys^8^) was slightly deeper within the hydrophobic core and the N-terminus was a little more exposed than the C-terminus (Figure 8C) as previously observed with MD simulation performed in neutral DPC micelle (Sani et al., 2020). In the bilayer system, the peptide maintained a transmembrane orientation (Figure 8B), with residues 6-18 significantly shielded from water exposure. Interestingly, some water molecules were found to reside for a notable amount of time near Lys^8^ (side chain), His^12^ and Ala^16^ (Figure 8D). A snapshot showed Mac1 with a bent structure and ^8^Lys extending to reach the phosphate-water interface and water flowing along the hydrophilic i,i+4 side of the peptide as shown in Figure 1E. Note, however, that the simulations performed herein were with an inserted peptide but earlier MD results (Wang et al., 2016) show that Mac1 inserts transmembrane into a phospholipid bilayer from the aqueous phase.

#### Peptide Pairing With Anionic vs. Neutral Lipids

**Figure 9:**
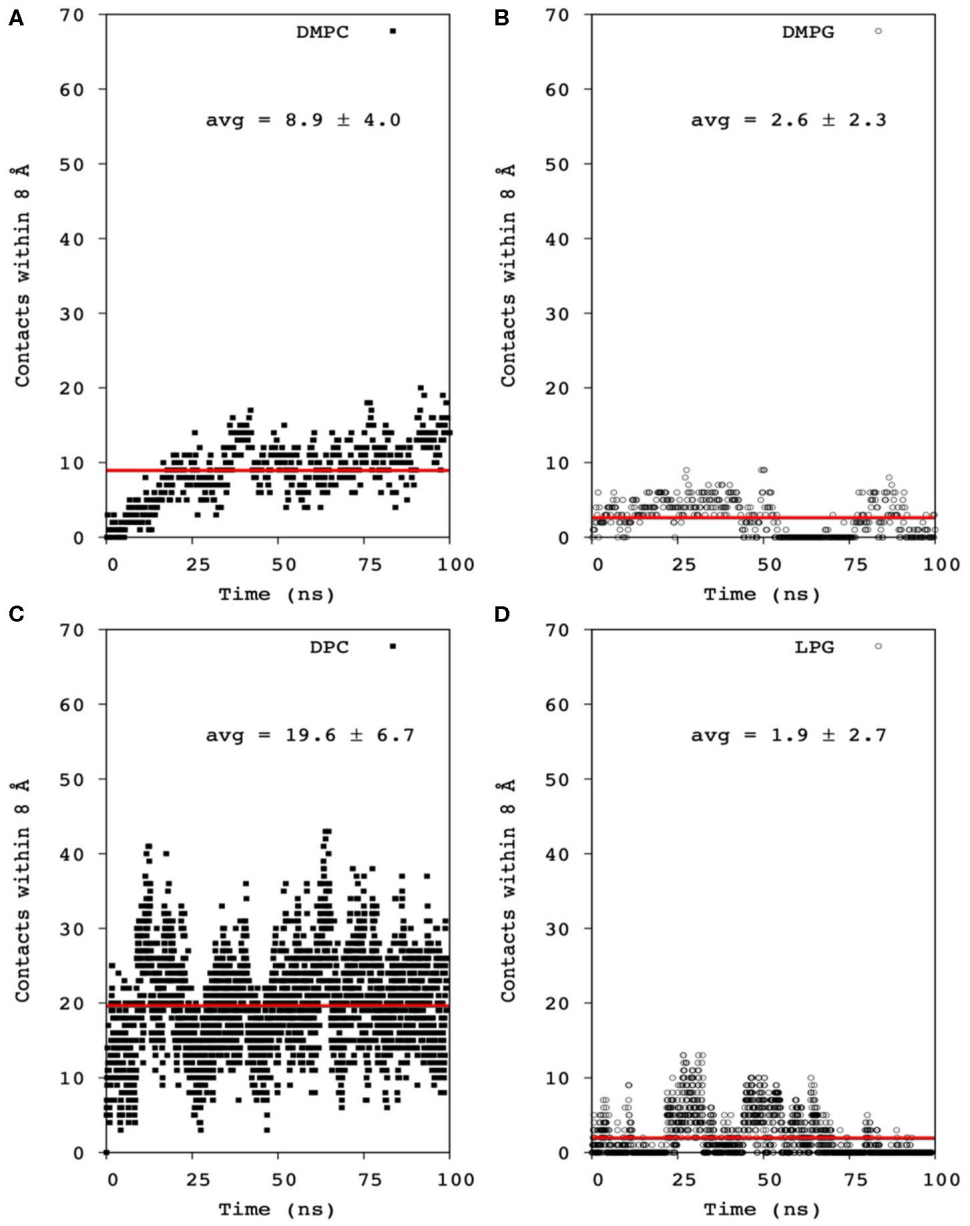
Averaged contact number within 8 Å between all Mac1 Cα atoms and phosphates of **(A)** DMPC and nitrogen atoms, **(B)** DMPG and hydroxyl atoms, **(C)** DPC and nitrogen atoms, and **(D)** LMPG and hydroxyl atoms. The averaged contact number was obtained by fitting the data to a linear function (red line).

Mac1 is a cationic peptide, but with a rather low overall charge of +1 at neutral pH. It has been shown to interact with many types of lipid membranes, inducing severe leakage in neutral and anionic membranes, but to a greater extent in the former (Fernandez et al., 2013a). However, electrostatic attractions have been shown to dominate the first binding step, as competition between neutral and anionic lipids have a larger preference to the latter surface. Nonetheless, once bound and inserted within the lipid hydrophobic core, MD simulations showed that the lipid headgroup has little influence on the peptide-lipid pairing as no segregation was observed within the simulation timeframe (Figure 9). Several possibilities are to be considered: (1) the simulation was not long enough to reach a pairing equilibrium; (2) Mac1 is mainly located below the phosphate headgroup and thus less prone to sense the headgroup difference between phosphocholine and phosphoglycerol moieties; and 3) the density of anionic lipids is not sufficient for statistically significant pairing to occur.

## Discussion

Previous studies investigating the location and orientation of Mac1 within phospholipid membranes focused on using zwitterionic PC headgroups (Sani et al., 2020), which generally model most eukaryotic cell membranes well, but do not replicate the anionic nature of bacterial membranes which are rich in PG and other anionic lipids. Whilst knowing how AMPs behave in zwitterionic membranes environments is important as it has to be understood how host cells are going to be affected by AMP treatment, ultimately it is the interactions with the bacterial pathogen that are of interest and this work has addressed the structure of the peptide-lipid aggregates that form. We find that the secondary structure of Mac1 remains unchanged whether in a zwitterionic lipid or an anionic environment (Figure 1). This suggests that headgroup composition plays little or no role in Mac1 structure once embedded within a membrane and that, as previous studies have shown, tail composition is the important factor in driving Mac1 conformation when embedded within a membrane (Sani et al., 2012). In addition, Mac1 still embeds deeply into the membrane in the presence of anionic lipids and assembles in a parallel fashion as observed for zwitterionic membranes (Sani et al., 2020). However, with zwitterionic membranes and the use of d-Mac1 it was observed that Mac1 adopts a preferred orientation with the N-terminus in the outer leaflet of the membrane (Sani et al., 2020). In this work, however, a preferred orientation within anionic membranes could not be conclusively determined in solid-supported membranes as the difference in SLD between the tails in each leaflet was with within error of each other (Figure 4 and Supplementary Table S5). This does not mean that a preferred orientation is ruled out as a preferred orientation in this case can be masked either due to the greater solvent content observed with d-Mac1 bound to anionic membranes, or some uneven distribution of the peptide across the bilayer leaflets. The location of d-Mac1 also was not influenced by the phase of the membrane (Figure 5).

Mac1 orientation was inconclusive in solid-supported anionic bilayers with the difference in SLD either being due to a uneven distribution of peptide across the different leaflets or parallel assembly with a preferential orientation. Other studies have examined the orientation of Mac1 within a phopholipid bilayer and show a variety of orientations based on different lipid compositions and model membranes (Chia et al., 2002; Bond et al., 2008; Wang et al., 2016), with a defined orientation being inconclusive for anionic bilayers (Balatti et al., 2018). However, in each case Mac1 spans the bilayer and, given that the volume fractions between each leaflet in the solid-supported bilayers are within error of each other (Supplementary Table S4) and the MD simulations suggest a similar orientation as for zwitterionic membranes with the N-terminus more exposed to water (Figure 8), it is likely that in this case d-Mac1 also spans the anionic bilayer. Where previous MD work has shown uneven distribution of Mac1 across the leaflets of a planar bilayer (Bond et al., 2008; Balatti et al., 2018), the difference in distribution is small (<15% difference) and these small difference would not greatly impact on the NR results, further supporting a membrane spanning model. Interestingly, the helical structure of Mac1 generates a hydrophilic surface, which in addition to Lys^8^ snorkeling out to reach the water interface, allowed water to penetrate deeply into the bilayer hydrophobic core (Figures 7, 8). This may be the initial step for promoting peptide-peptide self-assembly that would form larger pore structures, as previously demonstrated experimentally (Sani et al., 2013) and in μs-long MD simulations (Wang et al., 2016).

It has been previously shown that membranes containing anionic lipids bind more peptide molecules per lipid (Mechler et al., 2007; Fernandez et al., 2013a), despite neutral membranes being more prone to lytic activity (Sani et al., 2014), and this work is no exception to that. However, what is shown here is that anionic lipids are important in their preferential binding to cationic Mac1. Figure 2 demonstrates that Mac1 has a preference for anionic lipids and has little impact on zwitterionic lipids in a mixed phospholipid bilayer. Possibly the presence of Mac1 causes the PC and PG headgroups to laterally separate (Fernandez et al., 2013b) but the techniques used in this work do not demonstrate this, which is the subject of future studies.

There is a wide range of AMPs that target anionic lipids either for entry into a bacterial cell to reach its final target or because the mode of action is membrane disruption (Epand and Epand, 2009). How different AMPs target and assemble in bacterial membranes varies greatly depending on the peptide and the lipids used. In the case of indolicidin, when bound to model bacterial membranes the distribution of lipids and the membrane thickness are unaffected (Nielsen et al., 2018). Whereas, the short 13 amino acid peptide aurein 1.2, which operates through the carpet mechanism causes lateral segregation of mixed lipid systems (Sharma and Qian, 2019). Our results show that for the case of Mac1 when presented to a phospholipid bilayer of mixed anionic and zwitterionic lipids, the AMP preferentially assembles in a transmembrane fashion and targets the anionic lipids.

In conclusion, Mac1 embeds itself within a model bacterial membrane in a fashion that spans the whole membrane. The presence of anionic lipids does not dramatically alter the overall mechanism of Mac1 compared to membranes composed entirely of zwitterionic lipids; however, the presence of anionic lipids does attract Mac1 to the membrane which allows the peptide to better target membranes from pathogenic bacteria over host membranes.

## Data Availability Statement

The raw data supporting the conclusions of this article will be made available by the authors, without undue reservation.

## Author Contributions

AL, SZ, and M-AS conducted the experiments and the data analysis. FS conceived and lead the work. All authors wrote the paper.

## Conflict of Interest

The authors declare that the research was conducted in the absence of any commercial or financial relationships that could be construed as a potential conflict of interest.

## References

[B1] Balatti G. E. Martini M. F. Pickholz M. (2018). A coarse-grained approach to studying the interactions of the antimicrobial peptides aurein 1.2 and maculatin 1.1 with POPG/POPE lipid mixtures. J. Mol. Model. 24, 208. 10.1007/s00894-018-3747-z 30019106

[B2] Balhara V. Schmidt R. Gorr S.-U. DeWolf C. (2013). Membrane selectivity and biophysical studies of the antimicrobial peptide GL13K. Biochim. Biophys. Acta 1828, 2193–2203. 10.1016/j.bbamem.2013.05.027 23747365

[B3] Bond P. J. Parton D. L. Clark J. F. Sansom M. S. P. (2008). Coarse-grained simulations of the membrane-active antimicrobial peptide maculatin 1.1. Biophys. J. 95, 3802–3815. 10.1529/biophysj.108.128686 18641064 PMC2553143

[B4] Brogden K. A. (2005). Antimicrobial peptides: pore formers or metabolic inhibitors in bacteria? Nat. Rev. Microbiol. 3, 238–250. 10.1038/nrmicro1098 15703760

[B5] Chen V. B. Arendall W. B. III Headd J. J. Keedy D. A. Immormino R. M. Kapral G. J.. (2010). MolProbity: all-atom structure validation for macromolecular crystallography. Acta Crystallogr. D 66, 12–21. 10.1107/S0907444909042073 20057044 PMC2803126

[B6] Cheung M.-S. Maguire M. L. Stevens T. J. Broadhurst R. W. (2010). DANGLE: A Bayesian inferential method for predicting protein backbone dihedral angles and secondary structure. J. Magn. Reson. 202, 223–233. 10.1016/j.jmr.2009.11.008 20015671

[B7] Chia B. C. S. Carver J. A. Mulhern T. D. Bowie J. H. (2000). Maculatin 1.1, an anti-microbial peptide from the Australian tree frog, Litoria genimaculata - Solution structure and biological activity. Eur. J. Biochem. 267, 1894–1908. 10.1046/j.1432-1327.2000.01089.x 10727928

[B8] Chia C. S. B. Torres J. Cooper M. A. Arkin I. T. Bowie J. H. (2002). The orientation of the antibiotic peptide maculatin 1.1 in DMPG and DMPC lipid bilayers. Support for a pore-forming mechanism. FEBS Lett. 512, 47–51. 10.1016/S0014-5793(01)03313-0 11852050

[B9] Epand R. M. Epand R. F. (2009). Lipid domains in bacterial membranes and the action of antimicrobial agents. Biochim. Biophys. Acta 1788, 289–294. 10.1016/j.bbamem.2008.08.023 18822270

[B10] Fernandez D. I. Gehman J. D. Separovic F. (2009). Membrane interactions of antimicrobial peptides from Australian frogs. Biochim. Biophys. Acta 1788, 1630–1638. 10.1016/j.bbamem.2008.10.007 19013126

[B11] Fernandez D. I. Le Brun A. P. Lee T.-H. Bansal P. Aguilar M.-I. James M.. (2013a). Structural effects of the antimicrobial peptide maculatin 1.1 on supported lipid bilayers. Eur. Biophys. J. 42, 47–59. 10.1007/s00249-012-0796-6 22354331

[B12] Fernandez D. I. Lee T.-H. Sani M.-A. Aguilar M.-I. Separovic F. (2013b). Proline facilitates membrane insertion of the antimicrobial peptide maculatin 1.1 via surface indentation and subsequent lipid disordering. Biophys. J. 104, 1495–1507. 10.1016/j.bpj.2013.01.059 23561526 PMC3617439

[B13] Heinrich F. Ng T. Vanderah D. J. Shekhar P. Mihailescu M. Nanda H.. (2009). A new lipid anchor for sparsely tethered bilayer lipid membrane. Langmuir 25, 4219–4229. 10.1021/la8033275 19714901

[B14] Holt S. A. Le Brun A. P. Majkrzak C. F. McGillivray D. J. Heinrich F. Loesche M.. (2009). An ion-channel-containing model membrane: structural determination by magnetic contrast neutron reflectometry. Soft Matter. 5, 2576–2586. 10.1039/b822411k 21311730 PMC3035324

[B15] Humphrey W. Dalke A. Schulten K. (1996). VMD: Visual molecular dynamics. J. Mol. Graph. 14, 33–38. 10.1016/0263-7855(96)00018-5 8744570

[B16] Izaguirre J. A. Catarello D. P. Wozniak J. M. Skeel R. D. (2001). Langevin stabilization of molecular dynamics. J. Chem. Phys. 114, 2090–2098. 10.1063/1.1332996

[B17] James M. Nelson A. Holt S. A. Saerbeck T. Hamilton W. A. Klose F. (2011). The multipurpose time-of-flight neutron reflectometer “Platypus” at Australia's OPAL reactor. Nucl. Instrum. Meth. A 632, 112–123. 10.1016/j.nima.2010.12.07522938267

[B18] Jiang Z. Vasil A. I. Hale J. D. Hancock R. E. W. Vasil M. L. Hodges R. S. (2008). Effects of net charge and the number of positively charged residues on the biological activity of amphipathic α-helical cationic antimicrobial peptides. Pept. Sci. 90, 369–383. 10.1002/bip.20911 18098173 PMC2761230

[B19] Jo S. Kim T. Im W. (2007). Automated builder and database of protein/membrane complexes for molecular dynamics simulations. PLoS ONE 2:e880. 10.1371/journal.pone.0000880 17849009 PMC1963319

[B20] Jo S. Kim T. Iyer V. G. Im W. (2008). CHARMM-GUI: a web-based graphical user interface for CHARMM. J. Comput. Chem. 29, 1859–1865. 10.1002/jcc.20945 18351591

[B21] Jo S. Lim J. B. Klauda J. B. Im W. (2009). CHARMM-GUI membrane builder for mixed bilayers and its application to yeast membranes. Biophys. J. 97, 50–58. 10.1016/j.bpj.2009.04.013 19580743 PMC2711372

[B22] Koehbach J. Craik D. J. (2019). The vast structural diversity of antimicrobial peptides. Trends Pharmacol. Sci. 40, 517–528. 10.1016/j.tips.2019.04.012 31230616

[B23] Kyte J. Doolittle R. F. (1982). A simple method for displaying the hydropathic character of a protein. J. Mol. Biol. 157, 105–132. 10.1016/0022-2836(82)90515-0 7108955

[B24] Lakey J. H. (2019). Recent advances in neutron reflectivity studies of biological membranes. Curr. Opin. Colloid Interface Sci. 42, 33–40. 10.1016/j.cocis.2019.02.012

[B25] Lee D.-K. Brender J. R. Sciacca M. F. M. Krishnamoorthy J. Yu C. Ramamoorthy A. (2013). Lipid composition-dependent membrane fragmentation and pore-forming mechanisms of membrane disruption by pexiganan (MSI-78). Biochemistry 52, 3254–3263. 10.1021/bi400087n 23590672 PMC3795814

[B26] Lee T.-H. Heng C. Separovic F. Aguilar M.-I. (2014). Comparison of reversible membrane destabilisation induced by antimicrobial peptides derived from Australian frogs. Biochim. Biophys. Acta 1838, 2205–2215. 10.1016/j.bbamem.2014.02.017 24593995

[B27] Lee T.-H. Hofferek V. Separovic F. Reid G. E. Aguilar M.-I. (2019). The role of bacterial lipid diversity and membrane properties in modulating antimicrobial peptide activity and drug resistance. Curr. Opin. Chem. Biol. 52, 85–92. 10.1016/j.cbpa.2019.05.025 31260926

[B28] Lee T.-H. Sani M.-A. Overall S. Separovic F. Aguilar M.-I. (2018). Effect of phosphatidylcholine bilayer thickness and molecular order on the binding of the antimicrobial peptide maculatin 1.1. Biochim. Biophys. Acta 1860, 300–309. 10.1016/j.bbamem.2017.10.007 29030245

[B29] Lee T. H. Hall K. N. Aguilar M. I. (2015). Antimicrobial peptide structure and mechanism of action: a focus on the role of membrane structure. Curr. Top. Med. Chem. 16, 25–39. 10.2174/1568026615666150703121700 26139112

[B30] Lewis R. N. A. H. Zhang Y.-P. McElhaney R. N. (2005). Calorimetric and spectroscopic studies of the phase behavior and organization of lipid bilayer model membranes composed of binary mixtures of dimyristoylphosphatidylcholine and dimyristoylphosphatidylglycerol. Biochim. Biophys. Acta 1668, 203–214. 10.1016/j.bbamem.2004.12.007 15737331

[B31] Marcotte I. Wegener K. L. Lam Y.-H. Chia B. C. S. de Planque M. R. R. Bowie J. H.. (2003). Interaction of antimicrobial peptides from Australian amphibians with lipid membranes. Chem. Phys. Lipids 122, 107–120. 10.1016/S0009-3084(02)00182-2 12598042

[B32] Mechler A. Praporski S. Atmuri K. Boland M. Separovic F. Martin L. L. (2007). Specific and selective peptide-membrane interactions revealed using quartz crystal microbalance. Biophys. J. 93, 3907–3916. 10.1529/biophysj.107.116525 17704161 PMC2084233

[B33] Miyamoto S. Kollman P. A. (1992). Settle: An analytical version of the SHAKE and RATTLE algorithm for rigid water models. J. Comput. Chem. 13, 952–962. 10.1002/jcc.540130805

[B34] Naumann C. Brumm T. Bayerl T. M. (1992). Phase transition behavior of single phosphatidylcholine bilayers on a solid spherical support studied by DSC, NMR and FT-IR. Biophys. J. 63, 1314–1319. 10.1016/S0006-3495(92)81708-3 19431855 PMC1261435

[B35] Nelson A. (2006). Co-refinement of multiple-contrast neutron/X-ray reflectivity data using MOTOFIT. J. Appl. Crystallogr. 39, 273–276. 10.1107/S0021889806005073

[B36] Nelson A. (2010). Motofit – integrating neutron reflectometry acquisition, reduction and analysis into one, easy to use, package. J. Phys. Confer. Series 251:012094 10.1088/1742-6596/251/1/012094

[B37] Nielsen J. E. Bjørnestad V. A. Lund R. (2018). Resolving the structural interactions between antimicrobial peptides and lipid membranes using small-angle scattering methods: the case of indolicidin. Soft Matter 14, 8750–8763. 10.1039/C8SM01888J 30358793

[B38] Penfold J. Thomas R. K. (2014). Neutron reflectivity and small angle neutron scattering: An introduction and perspective on recent progress. Curr. Opin. Colloid Interface Sci. 19, 198–206. 10.1016/j.cocis.2014.01.002

[B39] Rice L. B. (2009). The clinical consequences of antimicrobial resistance. Curr. Opin. Microbiol. 12, 476–481. 10.1016/j.mib.2009.08.001 19716760

[B40] Rieping W. Habeck M. Bardiaux B. Bernard A. Malliavin T. E. Nilges M. (2006). ARIA2: Automated NOE assignment and data integration in NMR structure calculation. Bioinformatics 23, 381–382. 10.1093/bioinformatics/btl589 17121777

[B41] Roe D. R. Cheatham T. E. (2013). PTRAJ and CPPTRAJ: Software for processing and analysis of molecular dynamics trajectory data. J. Chem. Theory Comput. 9, 3084–3095. 10.1021/ct400341p 26583988

[B42] Rozek T. Waugh R. J. Steinborner S. T. Bowie J. H. Tyler M. J. Wallace J. C. (1998). The maculatin peptides from the skin glands of the tree frog Litoria genimaculata: a comparison of the structures and antibacterial activities of maculatin 1.1 and caerin 1.1. J. Pept. Sci. 4, 111–115. 10.1002/(SICI)1099-1387(199804)4:2<111::AID-PSC134>3.0.CO;2-8 9620615

[B43] Ryckaert J.-P. Ciccotti G. Berendsen H. J. C. (1977). Numerical integration of the cartesian equations of motion of a system with constraints: molecular dynamics of n-alkanes. J. Comput. Phys. 23, 327–341. 10.1016/0021-9991(77)90098-5

[B44] Sani M.-A. Gagne E. Gehman J. D. Whitwell T. C. Separovic F. (2014). Dye-release assay for investigation of antimicrobial peptide activity in a competitive lipid environment. Eur. Biophys. J. 43, 445–450. 10.1007/s00249-014-0970-0 24906225

[B45] Sani M.-A. Henriques S. T. Weber D. Separovic F. (2015a). Bacteria may cope differently from similar membrane damage caused by the australian tree frog antimicrobial peptide maculatin 1.1. J. Biol. Chem. 290, 19853–19862. 10.1074/jbc.M115.643262 26100634 PMC4528145

[B46] Sani M.-A. Le Brun A. P. Separovic F. (2020). The antimicrobial peptide maculatin self assembles in parallel to form a pore in phospholipid bilayers. Biochim. Biophys. Acta 1862:183204. 10.1016/j.bbamem.2020.183204 31981588

[B47] Sani M.-A. Lee T.-H. Aguilar M.-I. Separovic F. (2015b). Proline-15 creates an amphipathic wedge in maculatin 1.1 peptides that drives lipid membrane disruption. Biochim. Biophys. Acta 1848(10 Part A), 2277–2289. 10.1016/j.bbamem.2015.06.013 26079051

[B48] Sani M.-A. Separovic F. (2016). How membrane-active peptides get into lipid membranes. Acc. Chem. Res. 49, 1130–1138. 10.1021/acs.accounts.6b00074 27187572

[B49] Sani M.-A. Whitwell T. C. Separovic F. (2012). Lipid composition regulates the conformation and insertion of the antimicrobial peptide maculatin 1.1. Biochim. Biophys. Acta 1818, 205–211. 10.1016/j.bbamem.2011.07.015 21801711

[B50] Sani M. A. Whitwell T. C. Gehman J. D. Robins-Browne R. M. Pantarat N. Attard T. J.. (2013). Maculatin 1.1 disrupts Staphylococcus aureus lipid membranes via a pore mechanism. Antimicrob. Agents Chemother. 57, 3593–3600. 10.1128/AAC.00195-13 23689707 PMC3719708

[B51] Sharma V. K. Qian S. (2019). Effect of an antimicrobial peptide on lateral segregation of lipids: a structure and dynamics study by neutron scattering. Langmuir 35, 4152–4160. 10.1021/acs.langmuir.8b04158 30720281

[B52] Vranken W. F. Boucher W. Stevens T. J. Fogh R. H. Pajon A. Llinas M.. (2005). The CCPN data model for NMR spectroscopy: development of a software pipeline. Proteins Struct. Funct. Bioinformatics 59, 687–696. 10.1002/prot.20449 15815974

[B53] Wacklin H. P. (2010). Neutron reflection from supported lipid membranes. Curr. Opin. Colloid Interface Sci. 15, 445–454. 10.1016/j.cocis.2010.05.008

[B54] Wang Y. Chen C. H. Hu D. Ulmschneider M. B. Ulmschneider J. P. (2016). Spontaneous formation of structurally diverse membrane channel architectures from a single antimicrobial peptide. Nat. Commun. 7:13535. 10.1038/ncomms13535 27874004 PMC5121426

[B55] Wu E. L. Cheng X. Jo S. Rui H. Song K. C. Dávila-Contreras E. M.. (2014). CHARMM-GUI membrane builder toward realistic biological membrane simulations. J. Comput. Chem. 35, 1997–2004. 10.1002/jcc.23702 25130509 PMC4165794

