# Supplementary material for: The Location of the Antimicrobial Peptide Maculatin 1.1 in Model Bacterial Membranes
Source: Front Chem. 2020 Jul 7;8:572. doi: 10.3389/fchem.2020.00572 (PMC7358649; doi:10.3389/fchem.2020.00572)
Supplement: Supplementary file 1 [file Table_1.docx]

Supplementary Material

**The Location of the Antimicrobial Peptide Maculatin in Model Bacterial Membranes**

**Anton P. Le Brun^1^, Shiying Zhu^2^, Marc-Antoine Sani^2,*^ and Frances Separovic^2^**

^1^ Australian Centre for Neutron Scattering, Australian Nuclear Science and Technology Organisation, Kirrawee DC, NSW 2232, Australia

^2^ School of Chemistry, Bio21 Institute, University of Melbourne, Melbourne, VIC 3010, Australia

*** Correspondence:**Dr Marc-Antoine Sani
msani@unimelb.edu.au

# Supplementary Figures and Tables

## Supplementary Figures

**
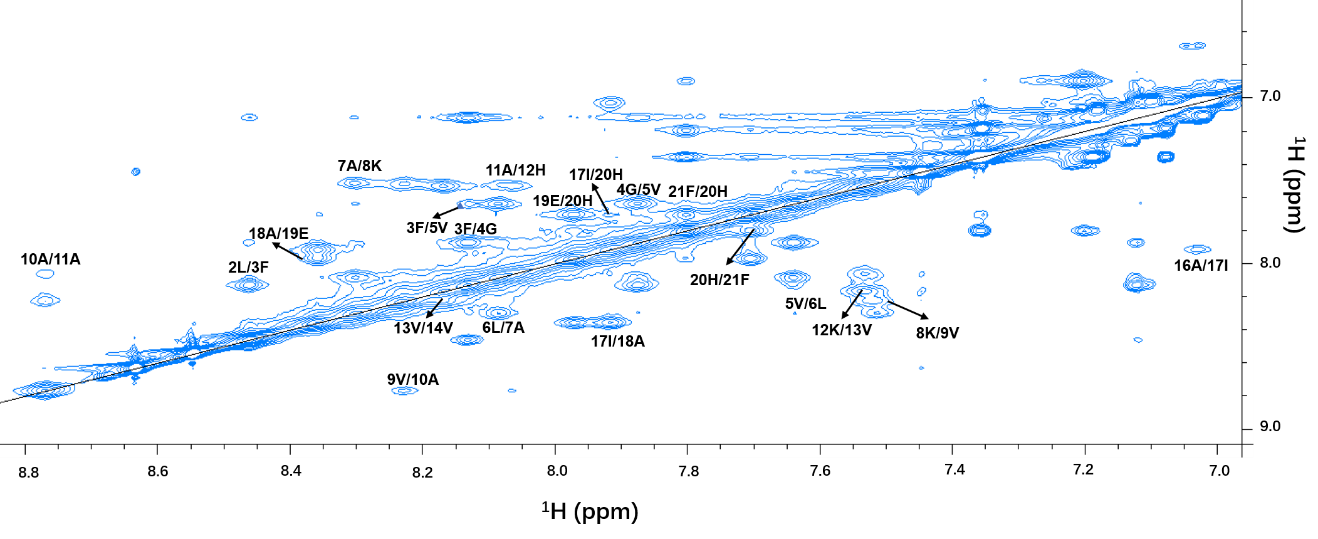
**

**Supplementary Figure S1:** Expanded NH region of the NOESY spectra (τ_mix_ = 150 ms) obtained from 3.5 mM Mac1 in 400 mM d_25_-SDS micelles (50 mM NaCl, 20 mM phosphate buffer, 0.5 mM NaN_3_, 0.03 mM TSP, 10%v D_2_O, pH=4.74) at 37ºC.

**Supplementary Figure S2:** Distances from the N^th^ residue to center of mass of DPC/LMPG (9:1) bilayer across 100 ns MD simulation. The dashed green lines correspond to 66^th^ linear fit of the distance distribution.

**Supplementary Figure S3:** Z-axis projected distances from the N^th^ residue to the Z-axis projected center of mass of DMPC/DMPG (4:1) bilayer across 100 ns MD simulation. The dashed green lines correspond to the Gaussian fit of the distance distribution.

**Supplementary Figure S4:** Number of contacts within 5 Å between Mac1 backbone nitrogen and water molecules in DPC/LMPG (9:1) across the 100 ns simulation. The dashed green lines correspond to the linear fit of the contact distribution.

**Supplementary Figure S5:** Number of contacts within 5 Å between Mac1 backbone nitrogen and water molecules in DMPC/DMPG (4:1) across the 100 ns simulation. The dash green lines correspond to the linear fit of the contact distribution.

## Supplementary Tables

**Supplementary Table S1:** Theoretical scattering length densities (SLD) for the materials used in the study.

| Component | Composition | SLD in D_2_O / 🞨10^-6^ Å^-2^ | SLD in H_2_O / 🞨10^-6^ Å^-2^ | Volume^#,^^ / Å^3^ | Number of exchangeable hydrogens |
| --- | --- | --- | --- | --- | --- |
| PC/PG (3:1) headgroups | (C_10_H_18_O_8_NP)_0.75_ (C_8_H_12_O_10_P)_0.25_ | 2.31 | 2.11 | 304 | 0.5 |
| H-lipid tails  (L_α_ phase) | C_26_H_54_ | -0.37 | -0.37 | 782 | 0 |
| H-lipid tails  (L_β_ phase) | C_26_H_54_ | -0.41 | -0.41 | 710 | 0 |
| d_54_-lipid tails  (L_α_ phase) | C_26_D_54_ | 6.82 | 6.82 | 782 | 0 |
| d_54_-lipid tails  (L_β_ phase) | C_26_D_54_ | 7.51 | 7.51 | 710 | 0 |
| d-Mac1* | GLFG**VLA**K**VAA**  HVVPAIAEHF-NH_2_ | 4.14 | 3.13 | 2775 | 27 |
| N-terminal d-Mac1* | GLFG**VLA**K**VAA** | 5.43 | 4.37 | 1369 | 14 |
| C-terminal d-Mac | HVVPAIAEHF-NH_2_ | 2.89 | 1.92 | 1406 | 13 |

*Residues in bold are deuterated.

^#^Volumes for lipids taken from references (Petrache *et al*., 1998; Tristram-Nagle *et al*., 2002; Pabst *et al.*, 2007).

^^^Volumes for amino acids taken from reference (Perkins, 1986).

**Supplementary Table S2:** Statistical information for the Mac1 NMR structures in SDS micelles

|  |  | Mac1 |
| --- | --- | --- |
| **Distance restraints** | Total | 170 |
|  | Intra residue | 94 |
|  | Inter | 76 |
|  | Sequential | 63 |
|  | Short range (seq) | 76 |
|  | Short range (non seq) | 13 |
| **Atomic RMSD (Å) from average structure** | | |
| Cα (1-21) | | 0.64 |
| **Violations from experimental restraints** | | |
| nOe violations (> 0.3 Å) | | 0 |
| Average violation (Å) | | 0 |
| **MolProbity statistics** | | |
| Clashes (> 0.4 Å / 1000 atoms) | | 9.9±2.88 |
| Ramachandran outliers (%) | | 0 |
| Ramachandran favoured (%) | | 95.7% |
| MolProbity score | | 1.8 |
| Cβ deviations>0.25 | | 0 |
| Bad bonds | | 0 |
| Bad angles | | 0 |

**Supplementary Table S3:** Fitted values for the solid supported phospholipid bilayers before addition of Mac1.

|  | 30°C | | | 15°C | | |
| --- | --- | --- | --- | --- | --- | --- |
|  | d_54_-DMPC/d_54_-DMPG (3:1) | | | | | |
|  | Thickness / Å | Lipid volume fraction | Area per lipid / Å^2^ | Thickness / Å | Lipid volume fraction | Area per lipid / Å^2^ |
| Inner headgroup | 15.2 ± 0.5 | 0.694 ± 0.050 |  | 16.1 ± 0.7 | 0.666 ± 0.051 |  |
| Tails | 30.5 ± 0.4 | 0.894 ± 0.134 | 57.4 ± 8.6 | 34.0 ± 0.4 | 0.920 ± 0.012 | 45.4 ± 0.8 |
| Outer headgroup | 6.3 ± 0.8 | 0.897 ± 0.415 |  | 14.6 ± 1.9 | 0.403 ± 0.034 |  |
|  | h-DMPC/h-DMPG (3:1) | | | | | |
| Inner headgroup | 14.7 ± 1.4 | 0.477 ± 0.049 |  | 13.7 ± 1.2 | 0.427 ± 0.045 |  |
| Tails | 33.1 ± 0.7 | 0.965 ± 0.030 | 49.0 ± 1.8 | 34.2 ± 0.7 | 0.923 ± 0.104 | 45.0 ± 5.2 |
| Outer headgroup | 11.6 ± 1.5 | 0.565 ± 0.084 |  | 14.6 ± 1.9 | 0.757 ± 0.279 |  |

**Supplementary Table S4:** Fitted results using a four layer model for the solid-supported phospholipid bilayers once d-Mac1 is bound.

|  | 30°C | | | 15°C | | |
| --- | --- | --- | --- | --- | --- | --- |
|  | d_54_-DMPC/d_54_-DMPG (3:1) + d-Mac1 | | | | | |
|  | Thickness / Å | Lipid volume fraction | Peptide volume fraction | Thickness / Å | Lipid volume fraction | Peptide volume fraction |
| Inner headgroup | 13.0 ± 2.2 |  |  | 15.1 ± 1.0 |  |  |
| Inner tails | 16.8 ± 0.6 | 0.803 ± 0.030 | 0.172 ± 0.064 | 17.8 ± 0.4 | 0.455 ± 0.099 | 0.400 ± 0.087 |
| Outer tails | 16.0 ± 0.6 | 0.763 ± 0.019 | 0.209 ± 0.005 | 17.2 ± 0.4 | 0.590 ± 0.011 | 0.344 ± 0.062 |
| Outer headgroup | 12.6 ± 2.1 |  |  | 13.2 ± 1.1 |  |  |
|  | h-DMPC/h-DMPG (3:1) + d-Mac1 | | | | | |
| Inner headgroup | 21.7 ± 0.1 |  |  | 15.0 ± 0.9 |  |  |
| Inner tails | 15.3 ± 0.5 | 0.744 ± 0.089 | 0.118 ± 0.014 | 16.7 ± 0.9 | 0.761 ± 0.157 | 0.104 ± 0.027 |
| Outer tails | 15.9 ± 0.5 | 0.758 ± 0.076 | 0.182 ± 0.033 | 16.7 ± 0.8 | 0.821 ± 0.255 | 0.108 ± 0.033 |
| Outer headgroup | 15.4 ± 0.3 |  |  | 11.8 ± 0.3 |  |  |

**Supplementary Table S5:** Fitted SLD values of the inner and outer tails of the solid-supported phospholipid bilayers under experimental conditions.

|  | d_54_-DMPC/d_54_-DMPG (3:1) | | | | h-DMPC/h-DMPG (3:1) | | | |
| --- | --- | --- | --- | --- | --- | --- | --- | --- |
|  | 30°C | | 15°C | | 30°C | | 15°C | |
|  | SLD in D_2_O / 🞨10^-6^ Å^-2^ | SLD in H_2_O / 🞨10^-6^ Å^-2^ | SLD in D_2_O / 🞨10^-6^ Å^-2^ | SLD in H_2_O / 🞨10^-6^ Å^-2^ | SLD in D_2_O / 🞨10^-6^ Å^-2^ | SLD in H_2_O / 🞨10^-6^ Å^-2^ | SLD in D_2_O / 🞨10^-6^ Å^-2^ | SLD in H_2_O / 🞨10^-6^ Å^-2^ |
| Inner Tails | 5.62 ± 0.21 | 5.45 ± 0.09 | 6.00 ± 0.13 | 5.00 ± 0.07 | 0.75 ± 0.09 | -0.20 ± 0.09 | 0.68 ± 0.14 | -0.25 ± 0.13 |
| Outer Tails | 5.56 ± 0.14 | 5.37 ± 0.08 | 5.55 ± 0.10 | 5.09 ± 0.09 | 0.49 ± 0.09 | 0.09 ± 0.13 | 0.29 ± 0.09 | -0.20 ± 0.14 |
| Difference | 0.06 | 0.08 | 0.45 | -0.09 | 0.26 | -0.29 | 0.39 | -0.05 |

# Supplementary References

Pabst, G., Danner, S., Karmakar, S., Deutsch, G., and Raghunathan, V.A. (2007). On the Propensity of Phosphatidylglycerols to Form Interdigitated Phases. *Biophysical Journal* 93(2)**,** 513-525. doi: <https://doi.org/10.1529/biophysj.106.101592>.

Perkins, S.J. (1986). Protein volumes and hydration effects. *European Journal of Biochemistry* 157(1)**,** 169-180. doi: <https://doi.org/10.1111/j.1432-1033.1986.tb09653.x>.

Petrache, H.I., Tristram-Nagle, S., and Nagle, J.F. (1998). Fluid phase structure of EPC and DMPC bilayers. *Chemistry and Physics of Lipids* 95(1)**,** 83-94. doi: <https://doi.org/10.1016/S0009-3084(98)00068-1>.

Tristram-Nagle, S., Liu, Y., Legleiter, J., and Nagle, J.F. (2002). Structure of Gel Phase DMPC Determined by X-Ray Diffraction. *Biophysical Journal* 83(6)**,** 3324-3335. doi: <https://doi.org/10.1016/S0006-3495(02)75333-2>.
